# Transtibial versus independent femoral tunnel drilling techniques for anterior cruciate ligament reconstruction: evaluation of femoral aperture positioning

**DOI:** 10.1186/s13018-022-03040-5

**Published:** 2022-03-18

**Authors:** Haitham K. Haroun, Maged M. Abouelsoud, Mohamed R. Allam, Mahmoud M. Abdelwahab

**Affiliations:** 1 Orthopedic Department, Faculty of Medicine, Ain Shams University, Al-Abbasya District, Cairo, Egypt; 2 El Demerdash Hospital, Ain-Shams University, Cairo, Egypt

**Keywords:** ACL reconstruction, Femoral aperture, Femoral tunnel drilling, Transtibial, Tibial independent

## Abstract

**Background:**

Femoral tunnel can be drilled through tibial tunnel (TT), or independent of it (TI) by out-in (OI) technique or by anteromedial (AM) technique. No consensus has been reached on which technique achieves more proper femoral aperture position because there have been evolving concepts in the ideal place for femoral aperture placement. This meta-analysis was performed to analyze the current literature comparing femoral aperture placement by TI versus TT techniques in ACL reconstruction.

**Methods:**

We performed a comprehensive systematic review and meta-analysis of English-language literature in PubMed, Cochrane, and Web of Science databases for articles comparing femoral aperture placement by TI versus TT techniques with aperture position assessed by direct measurement or by postoperative imaging, PXR and/or CT and/or MRI.

**Results:**

We included 55 articles with study population of 2401 knees of whom 1252 underwent TI and 1149 underwent TT techniques. The relevant baseline characteristics, whenever compared, were comparable between both groups. There was nonsignificant difference between TI and TT techniques in the distance from aperture center to footprint center and both techniques were unable to accurately recreate the anatomic footprint position. TI technique significantly placed aperture at more posterior position than TT technique. TI technique significantly lowered position of placed aperture perpendicular to Blumensaat’s line (BL) than TT technique, and modifications to TT technique had significant effect on this intervention effect. Regarding sagittal plane aperture placement along both AP anatomical axis and BL, there was nonsignificant difference between both techniques.

**Conclusion:**

Modifications to TT technique could overcome limitations in aperture placement perpendicular to BL. The more anterior placement of femoral aperture by TT technique might be considered, to some extent, a proper position according to recent concept of functional anatomical ACL reconstruction.

**Supplementary Information:**

The online version contains supplementary material available at 10.1186/s13018-022-03040-5.

## Introduction

Improper femoral aperture placement is the most common cause of anterior cruciate ligament (ACL) reconstruction failure or unsatisfactory outcomes [1]. The criteria of proper femoral aperture placement had changed overtime. First, surgeons aimed mainly to restore the anteroposterior stabilizing function of ACL by isometric positioning of femoral aperture creating vertical ACL graft [2]. With further anatomical and biomechanical studies, surgeons realized the two-bundle anatomy of the ACL and the specific role of its lower, more shallow fibers (posterolateral bundle) in its rotatory stabilizing function. Accordingly, surgeons attempted to restore the native femoral footprint by inserting ACL graft at the footprint center (average site of the ACL two bundle) “Anatomical single bundle ACL reconstruction” [2]. However, given that recent research demonstrated that the size, morphology, and site of this footprint are highly variable from person to person and that this footprint is usually absent especially in chronic ACL injury, there is no standard guidelines applicable to all individuals that tell the surgeon where to place the ACL femoral aperture and the ACL graft insertion site should be individualized [1]. Moreover, there has been a recent discussion over the past decade that divided ACL insertion into direct and indirect fibers. This recent concept of ACL anatomy may affect the determination of the ideal place for performing femoral aperture where the new ligament might be positioned within the ACL footprint in the most functional load bearing position where the direct fibers lie "Functional anatomical ACL reconstruction" [1].

The femoral tunnel can be drilled through the tibial tunnel (TT), or independent of it (TI) by the out-in (OI) technique or by the anteromedial transportal (AM) technique. In TT technique, the placement of the femoral tunnel is dependent on the tibial tunnel [3]. However, because the diameter of the endoscopic femoral offset guide aimer and the shaft of the acorn femoral reamer are smaller than the tibial tunnel diameter, there is reasonable corresponding degree of freedom to externally rotate the offset guide and place the femoral tunnel as low in the intercondylar notch as possible [4], whereas in TI techniques, the surgeon could choose freely where to place the femoral tunnel, regardless of the tibial tunnel. However, this does not ensure proper placement of femoral tunnel, as surgeon may choose inappropriate positions [3].

There are advantages and disadvantages to each technique. The TT technique is more familiar and allows an isometric position and easy graft passage. However, disadvantages include increased vertical and potentially nonanatomic tunnels, alongside posterior tibial tunnel placement. The advantages of AM technique include the anatomical positioning of the femoral tunnel and theoretically better rotational stability. However, this approach may increase the risks of iatrogenic damage to the medial femoral condylar cartilage and creating critically short tunnels. The OI technique has additional advantage of lower risk of posterior wall rupture. However, acute femoral tunnel angle and additional lateral skin incisions are major impediments [3].

Difference in femoral tunnel orientation between both techniques has been studied extensively in literature. However, femoral tunnel obliquity does not reflect graft obliquity exactly. Graft obliquity is affected primarily by intraarticular aperture location of the femoral and tibial tunnels [5].

The aim of this review is to evaluate femoral aperture positioning achieved by each technique and its relevance to the modern studies assessing the anatomical and biomechanical properties of ACL femoral insertion. In addition, we assessed the effect of each technique on the tibial aperture position.

## Methods

### Search methods

We performed a comprehensive literature search of the following databases from their inception dates to March 2021: PubMed, Cochrane Central Register of Controlled Trials [CENTRAL], and Web of Science. Searches were carried out in accordance with PRISMA (Preferred Reporting Items for Systematic Reviews and Meta-analyses) [6]. Before the literature search, the research protocol for this review was registered with the PROSPERO international prospective register of systematic reviews and published online under registration number CRD42019133505. The full search strategy is presented in Additional file 1. Three independent reviewers (M.M.A., M.R.A, and H.K.H.) conducted the search separately. We also searched the reference lists of the included studies for additional eligible articles.

### Criteria for considering studies for this review

The eligibility criteria for studies were as follows: studies directly comparing TT versus TI (AM or OI) femoral drilling techniques for femoral aperture placement with aperture position assessed by direct measurement or by postoperative imaging: PXR and/or CT and/or MRI and quantified by an appropriate method**.** Studies should report aperture position by a suitable statistic describing average and distribution, and sample numbers. Abstracts, case reports, and conference presentations were excluded. Only articles in English were included.

The eligibility criteria for participants were as follows: Human or cadaveric subjects, following single bundle ACL reconstruction in skeletally mature individuals**.**

### Data collection and analysis

#### Study selection

The selection of studies was performed by 2 independent investigators (M.M.A. and M.R.A) separately. Any disagreement was resolved by an arbiter (most senior, third author [H.K.H.]).

#### Data extraction and management

Data from included studies were independently extracted into spreadsheets by the 3 investigators. In case of any missing data in any study, we tried to contact the corresponding author.

### Assessment of risk of bias

Two reviewers (M.M.A. and M.R.A) independently appraised each article. Any disagreement was resolved by the arbiter. The revised and validated version of the Methodological Index for Non-randomized Studies (MINORS) scoring system was used [7]. In brief, the MINORS scoring system provides a method to assess bias, with a higher score indicative of less bias. The optimum score for comparative studies is 24.

### Statistical analysis

We provided a qualitative synthesis of the findings from the included studies, structured according to the imaging technique and measurement method. If enough comparative studies were provided (at least 2) using the same measurement tool on the same imaging modality, a meta-analysis was performed. When trials included multiarm interventions, we combined arms utilizing the same intervention. A fixed-effects meta-analysis was used for combining data where it was reasonable to assume that studies were estimating the same underlying treatment effect. If substantial statistical heterogeneity (*I*^2^ statistics > 75%) was detected, the possible clinical and methodological reasons for this were explored qualitatively and quantitatively, also a random-effect model was used for meta-analysis. To quantitatively explore heterogeneity, we performed subgroup analyses searching for potential effect modification. Assessed effect modifiers included the type of experimental intervention (AM vs OI tibial independent technique) and modifications to the comparison intervention (conventional vs modified TT technique). Sensitivity analyses was performed to assess the effect of including studies of low-quality design (observational studies). We restricted these to femoral aperture position perpendicular to BL. We performed these analyses using RevMan software (version 5.3.5; Nordic Cochrane Centre, Copenhagen, Denmark).

## Results

### Search results

**Fig. 1:**
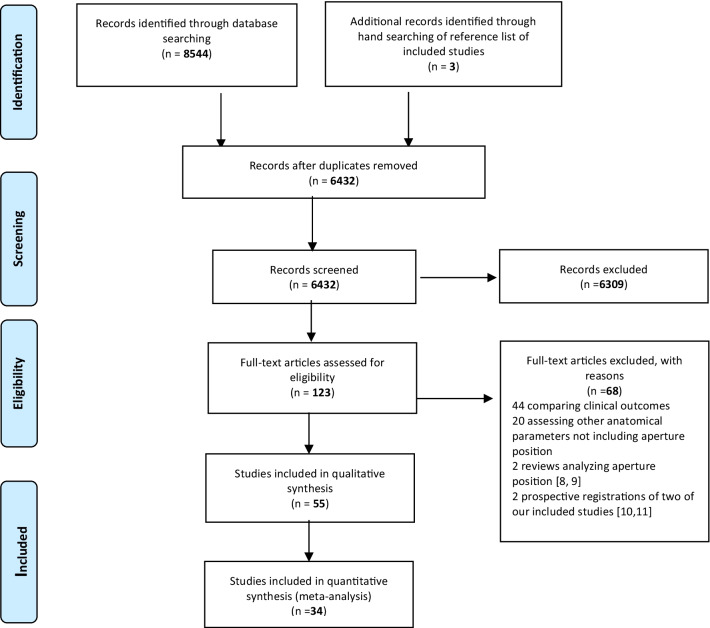
Flow diagram of methodology used for inclusion and exclusion of studies

Details of study identification, inclusion, and exclusion are shown in Fig. 1 [8–11].

### Study characteristics

Our review included 39 clinical studies [12–50] and 16 basic science studies [51–66]. The clinical studies included 21 retrospective cohort studies, 11 prospective cohort studies, and 7 randomized controlled trials (RCTs) [14, 24, 28, 36, 44, 48, 50]. (Three of them were registered, two [36, 50] prospectively in ClinicalTrials.gov and the third [24] retrospectively in Chinese Clinical Trial Registry.) From the 16 cadaveric studies, 6 were RCTs [53, 60–64] (2 of them quasi-randomized [53, 62]). Table in Additional file 2 summarizes the characteristics of included studies in this review, including number of knees assessed, imaging modality or its surrogate, and MINORS score for each study.

The design of all cadaveric studies was independent-measures design except 5 were conducted in a repeated-measures design [53, 54, 57, 58, 61]. The independent measures were on paired specimens in 6 studies [51, 55, 60, 62–64] (i.e., one knee of a cadaver pair was drilled with TT technique and the opposite knee was drilled with TI technique), on unpaired specimens in 4 studies [56, 59, 65, 66], and the pairing was unclear in one study [52].

### Participants characteristics

#### Clinical studies

From a total of 2401 knees, 1252 underwent TI femoral tunnel drilling whereas 1149 received TT drilling. The mean age of participants undergoing TI and TT drilling procedure was 30.9 and 29 years, respectively. In the 20 studies, in which gender was reported and could be calculated, the percentage of male patients who had undergone TI and the TT drilling procedure was 77% and 87%, respectively. The time between injury and surgery, whenever reported in 11 studies, varied from less than 3 months in 4 studies [24, 32, 36, 48] and more than three months in seven studies [13, 15, 16, 23, 28, 45, 49]. However, whenever compared [24, 32, 36, 48, 49], there was nonsignificant difference between both groups.

#### Cadaveric studies

In all studies, the specimens were fresh not embalmed thus preserving bony and soft tissue anatomy. From a total of 428 placed apertures (in 328 knees), 232 were placed by TI technique whereas 196 were placed by TT technique. The weighted average age of specimens, in the 11 studies reporting a known age, was 66.6 years. In the 3 studies, reporting a known specimen’s gender, there were 54 males, 14 females, and 2 undetermined genders. In the 6 studies reporting about articular cartilage quality of specimens [52, 54, 56, 60, 61, 65], there were no significant, or even no, arthritis. ACL was intact in the 8 studies reporting about its integrity [52, 54–58, 60, 61]

### Surgical techniques

#### Intervention integrity (the surgeon)

Thirty-five studies reported the number of surgeons which was same surgeon or surgical team in 22, two surgeons in 5, three or four surgeons in 5, eight to twenty-two surgeons in 3 studies. Most studies reported relevant information about surgeon's experience level like experience years or surgery volume or surgeon seniority. Qualitative inter-studies comparison showed that the surgeons had varying experience level. However, the type of the procedure chosen for each patient, in observational studies, was based on the preferred surgeon surgical technique.

#### Surgical technique of TT femoral tunnel drilling

From 47 studies reporting the intervention details, the TT technique included modifications in some of them. These modifications could be divided into (1) recreation of native coronal or sagittal ACL orientation by alteration of tibial tunnel extra-articular starting point in mediolateral (ML) or proximal-distal (PD) direction [67, 68], which was performed in 21 and 3 studies, respectively. (2) Alteration of position of tibial in relation to femur during femoral aperture placement. (3) AM portal-assisted TT femoral tunnel drilling. (4) Posterior "over the top" notchplasty (allowing increased offset guide rotation). (5) Intentional tibial aperture posterolateral beveling. As the first modification of altering tibial tunnel starting point is an old modification and was performed in nearly two-third (24/36) of studies reporting tibial tunnel starting point, in our review, modified technique (mTT) would denote only the other 4 modifications. So, there were 11 studies utilized mTT technique [14, 28, 32, 33, 47, 48, 50, 52, 57, 63, 64]. (Details of modifications in these studies are presented in table in Additional file 3).

The intraarticular target point for the tibial tunnel, whenever reported in 41 studies, had been aimed at the tibial footprint. However, it had been aimed at the anteromedial and posterolateral part of the footprint in 11 and 6 studies, respectively. From the 25 studies reporting tibial tunnel diameter, it was 8 mm, 9–10 mm, and 10–11 mm in 9, 4, and 12 studies, respectively. From the 47 studies reporting femoral aperture localization strategy, an offset femoral guide was utilized in 38 and was not utilized in 9 studies. Moreover, other utilized methods varied in different studies. (Details of localization strategies utilized in TT technique in included studies are presented in first table in Additional file 4).

#### Surgical technique of TI femoral tunnel drilling

Independent drilling of femoral tunnel was performed by AM technique in 37 studies or by OI technique in 10 studies. The TI technique was undetermined in one study [29]. In addition, in 7 three-arm studies, the independent drilling was performed by AM and OI techniques. From the 53 studies reporting femoral aperture localization strategy, the remnant ACL footprint was reported to be visualized in 18 studies. Also, the other utilized methods varied in different studies (Details of localization strategies utilized in TI technique in included studies are presented in second table in Additional file 4).

#### AM femoral tunnel drilling technique

From the 40 studies reporting intervention details of AM technique (total 44 studies), femoral tunnel was drilled through the single AM portal (two-portal technique) in 19 studies, through an accessory AM portal (three-portal technique) in 12 studies, and through either of them (in each of two subgroups) in one study [26]. The working AM portal was unclear in 6 studies [25, 38, 49, 50, 54, 61]. In 2 studies [56, 62], the femoral tunnel was drilled through medial parapatellar approach (open approach).

#### OI femoral tunnel drilling technique

From the 16 studies reporting intervention details of OI technique (total 17 studies), femoral tunnel was drilled by rear-entry guide in 3 studies [13, 15, 56] and front-entry guide in 13 studies.

### Quality assessment of included studies

From 47 studies reporting about conflicts of interest (COI), 18 studies had potential COI. Six studies judged to be of notable concern as they were funded by private company who could gain from the study results, e.g., manufacturer [12, 35, 50, 57, 58, 60].

The MINORS scoring system deemed studies as acceptable quality with low bias (Additional file 2). The mean MINORS score for all included studies was (965 points/55 studies) = 18.2 points {76% of total possible points; range, (62.5–100%)}.

Follow-up duration was defined as the time interval between surgery and timing of femoral aperture position assessment, with a shorter time interval (less than 3 to 6 months) associated with a lower risk of bias, due to tunnel widening being unlikely. From the 28 clinical studies reporting the follow-up period, femoral aperture was assessed within one month in 15 studies. In the remaining 12 studies the average follow-up duration was 13 months.

While the age, and its associated degenerative changes, could affect the anatomy of the footprint and even the notch, the gender could affect only assessment tools that quantify F aperture position by measuring distance in millimeter (mm) from anatomical landmark. In clinical studies, the age and gender, whenever compared in 27 clinical studies, were comparable between the 2 groups. In cadaveric studies, while independent-measures design, with measures performed on paired specimens, eliminated among-individuals variations (e.g., age, gender, OA) except side-to-side variation within the same individual, the repeated-measures design eliminated all among-participants variations. However, it could have a carryover effect. Precautions carried out in the repeated-measures design studies to eliminate this carryover effect included filling the created femoral tunnels with epoxy [57] or cement [53] after completion of the first femoral tunnel positioning technique. As aperture placement in the other 3 studies included wire placement procedure only (i.e., no tunnel), they had no carryover effect.

Regarding sample size of knees analyzed in each group, generally the sample size was somewhat higher in TI group as it was presented by 2 subgroups (AM&OI) in the 7 multiarm studies. Also, nine double arm studies had unbalanced size in their 2 groups.

Regarding intervention integrity, qualitative within-studies comparison showed that the surgeon's experience level was comparable between both groups. However, that comparability was uncertain in 3 studies [13, 30, 60] and in all studies with non-contemporary groups when the surgeon changed his technique from TT to TI femoral drilling (13 studies). Regarding surgical technique, in one study [62], there was a major performance bias of utilizing surgical navigation system for femoral aperture localization in TI group only. Also, in 4 studies [23, 39, 63, 64], anterior notchplasty was performed in TT group only.

### Effects of interventions (qualitative synthesis and meta-analysis)

**Fig. 2:**
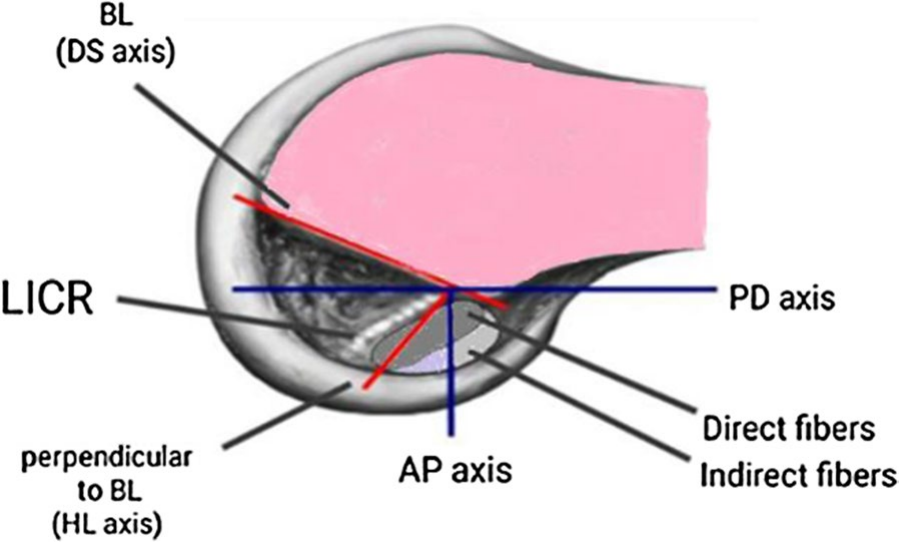
Schematic diagram of profile view of medial wall of lateral femoral condyle. LICR; lateral intercondylar ridge. Blue lines: Anatomical coordinates; PD (proximal-distal) and AP (antero-posterior) axes. Red lines: Blumensaat's line coordinates; BL (Blumensaat's line) or DS axis (deep-to-shallow axis) and perpendicular to BL or HL (high-to-low axis)

Comparison of femoral aperture placement between TI and TT technique was approached either directly by comparing the ability of each technique to create the anatomic footprint position or indirectly by comparing the quantified aperture location placed by each technique. Both aperture and footprint positions were defined in the coronal and sagittal planes. Coronal plane position was assessed along the anteroposterior (AP) anatomical axis, along the line perpendicular to the Blumensaat’s line (BL), and along mediolateral direction. Sagittal plane position was assessed along the PD anatomical axis and along BL (Fig. 2).

In addition, comparison of tibial aperture position between both techniques was performed whenever reported in included studies.

#### Direct approach of comparing the ability of each technique to create the anatomic footprint position

A. Footprint coronal plane position

1. Along AP anatomical axis

**Fig. 3:**
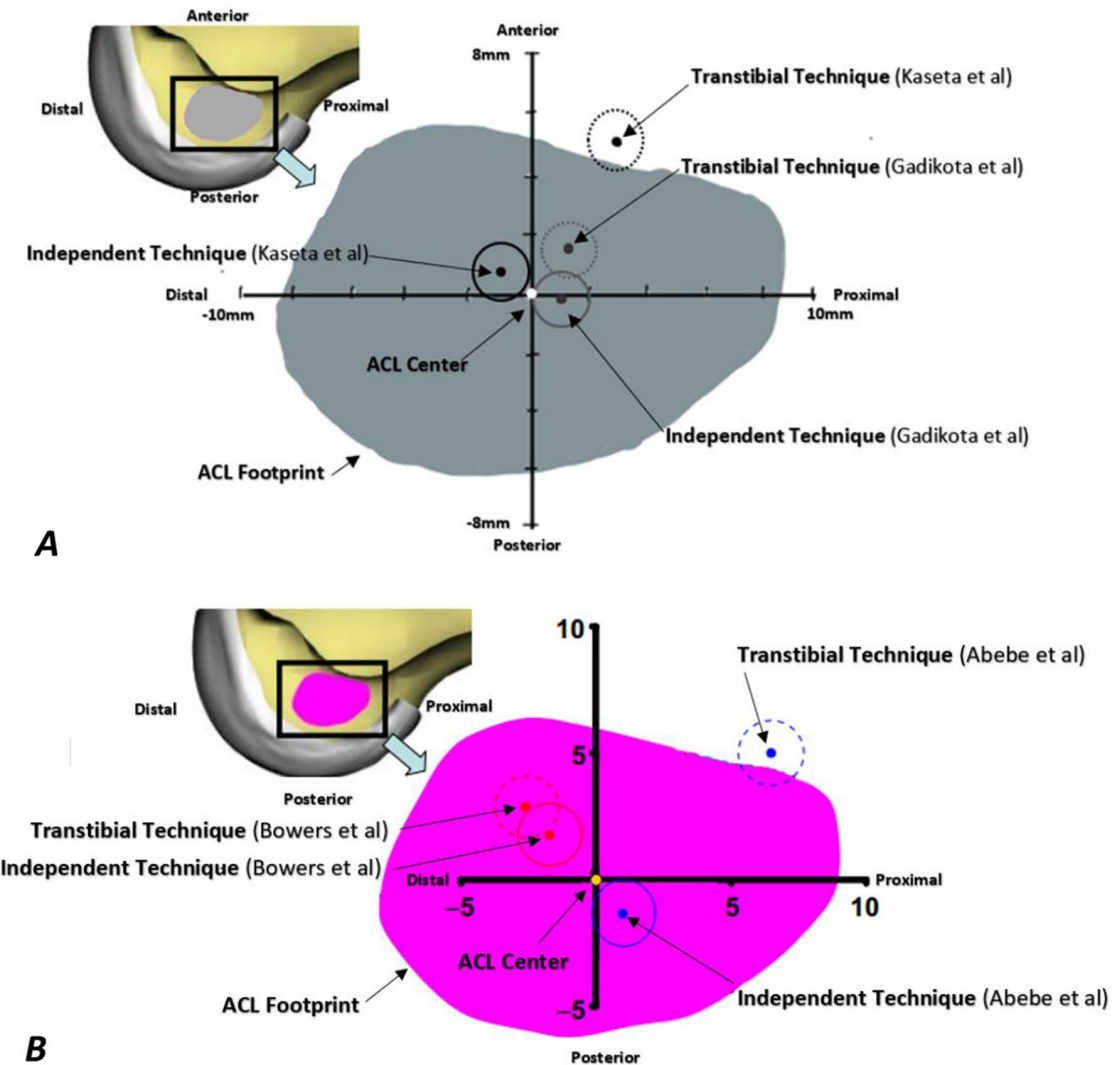
Visual display of the distance of aperture center to footprint center in postero-anterior and proximal-distal axes measured on digitized 3D model of same knee (**A**) and on MRI of contralateral knee (**B**). Each figure includes 2 dotted circles (transtibial) and 2 solid circles (independent technique) representing apertures placed in 2 studies that assessed the displayed outcome

Assessment of the distance of aperture center placed by each technique to the footprint center of the same knee on digitized 3d model in 2 studies (48 specimens) [53, 58] showed that TI technique placed aperture at less anterior distance from footprint than TT technique with a small mean difference of 3.3 mm (Additional file 5 and Fig. 3A). Also, while TT technique placed F aperture anterior to the footprint, albeit at small distance of 1.9 mm, TI technique placed it accurately at footprint center of same knee directly on 10 specimens (one study) [52]. Contradictory to the pervious findings, there was nonsignificant difference between both techniques in recreating the AP footprint position of the contralateral knee in 2 studies (46 participants) [12, 18] using MRI (Additional file 5 and Fig. 3B). Also, the difference in AP position%, measured by the anatomic coordinate axis (ACA) method, between aperture and footprint of contralateral knee, assessed on MRI, was nonsignificant between both techniques in one study (20 patients) [17].

2. Perpendicular to BL

In one study (20 specimens) [64], assessment of aperture spatial position in footprint referenced to BL on CT showed that TI technique placed a little bit more apertures in the lower deep quadrant of same knee footprint than TT technique. In another study [62]analyzing 20 specimens directly, TI technique placed significantly more apertures in the lower thirds of same knee footprint than TT. However, this study had a major performance bias of utilizing surgical navigation system for femoral aperture localization in TI group only.

B. Footprint sagittal plane position along PD anatomical axis

There was nonsignificant difference between both techniques in recreating the PD footprint position of the same knee assessed on digitized 3D model in two studies (48 specimens) [53, 58] (Additional file 5 and Fig. 3A). Consistent finding was demonstrated in 2 studies (46 participants) [18, 69] comparing aperture placed by each technique to the contralateral knee footprint on MRI (Additional file 5 and Fig. 3B). Contradictory to the previous findings, while TT technique placed F aperture proximal to the footprint, TI technique placed it accurately at footprint center of same knee directly assessed on 10 specimens (one study) [52]. Consistent finding was demonstrated in one study (20 participants) [17] assessing the difference in PD position, measured by ACA method, between aperture and footprint of the contralateral knee as measured on MRI.

III. Footprint absolute position

Directly comparing the distance of aperture center placed by each technique to footprint center of the same knee on digitized 3d model showed nonsignificant difference in 2 studies (48 specimens) [53, 58] (Additional file 5 and Fig. 3A). In 3 studies (87 patients) [12, 18, 26] assessing the ability of each technique to recreate the footprint position of the contralateral knee on MRI, there was nonsignificant difference in the distance of aperture center to footprint center (Additional file 5). Contradictory to the pervious findings, aperture placed by TI technique was significantly closer, albeit by small mean difference of 2.4 mm and 4 mm, in two studies (59 specimens) [61, 64] using CT scan. Assessing the ability of each technique to place the aperture center within the margins of footprint of same knee on photographed arthroscopic image in one study (20 specimens) [54], TI technique placed F aperture center at significantly closer distance to the closest point of footprint, albeit with a small mean difference of 3.4 mm. However, the tibial tunnel starting point in TT technique of this study was conventional point of Morgan [70]. For more detailed data, see summary of findings table of direct methods (Table 1).

**Table 1:** Summary of findings table of direct outcomes

Outcome	Illustrative comparative risks		No of participants (studies)
	Assumed risk	Corresponding risk (95% CI)	
*On same knee*			
Distance of aperture to FP			
On photographed arthroscopic image On digitized 3d model of specimen On CT	6.2 mm 5.9 mm NE	3.4 mm closer (3.6 mm closer:3.2 mm closer) 3.6 mm closer (8.3 mm closer:1.1 mm further) NE^b^	20 (1 study) 48 (2 studies^a^) 59 (2 studies)
Distance of aperture to FP in PA axis			
On specimen On digitized 3d model	1.9 mm anterior 3.7 mm anterior	1.9 mm more posterior 3.3 mm more posterior (6.2 mm more posterior:0.3 mm more posterior)	10 (1 study) 48 (2 studies)
Distance of aperture to FP in PD axis			
On specimen On digitized 3d model	3.3 mm proximal 2.9 mm proximal	3.3 mm more distal 2.9 mm more distal (6.1 mm more distal:0.3 mm more proximal)	10 (1 study) 48 (2 studies)
Aperture spatial position in FP			
On specimen On CT	100% in highest third Variable, 50% in lower deep quadrant	30% in highest, 50% in middle, and 20% in lower third Consistent, 70% in lower deep quadrant	20 (1 study) 20 (1 study)
*On contralateral knee on MRI*			
Greatest distance of aperture to FP	4 mm	1.3 mm closer (6 mm closer:3.4 mm further)	87 (3 studies)
Distance of aperture to FP in PA axis	3.7 mm anterior	3.5 mm more posterior (8.2 mm more posterior:1.2 mm more anterior)	46 (2 studies)
Distance of aperture to FP in DP axis	0.4 mm proximal	1.6 mm more distal (6 mm more distal:2.9 mm more proximal)	46 (2 studies)
Difference in AP position% between aperture and FP	9% anterior	2% more posterior (5% more anterior: 9% more posterior)	20 (1 study)
Difference in DP position% between aperture and FP	9% proximal	10% more distal (18.9% more distal:1.1% more distal)	20 (1 study)

FP, footprint; NE, not estimable

^a^A third study (72 participant) [57] investigating a substantial modification of TT technique (hybrid TT subgroup) whose result could not be pooled, showed 10 mm assumed risk (of conventional TT subgroup) and corresponding risk of 7.9 mm closer (10.5 mm closer to 5.3 mm closer)

^b^Qualitative synthesis: The results were consistent, both studies [51, 64] found that the TI technique placed aperture closer to the footprint than did the TT technique with mean difference of 2.4 mm and 4 mm

#### Indirect approach of comparing the quantified aperture location placed by TT and TI techniques

The aperture location was quantified either as percentage ratio of overall scaling dimension from the distal femur or as distance in millimeters (mm) from a fixed anatomic landmark.

A. Coronal plane position

1. Perpendicular to BL as percentage ratio of an overall scaling dimension from the lateral femoral condyle (LFC) or the intercondylar notch

**Fig. 4:**
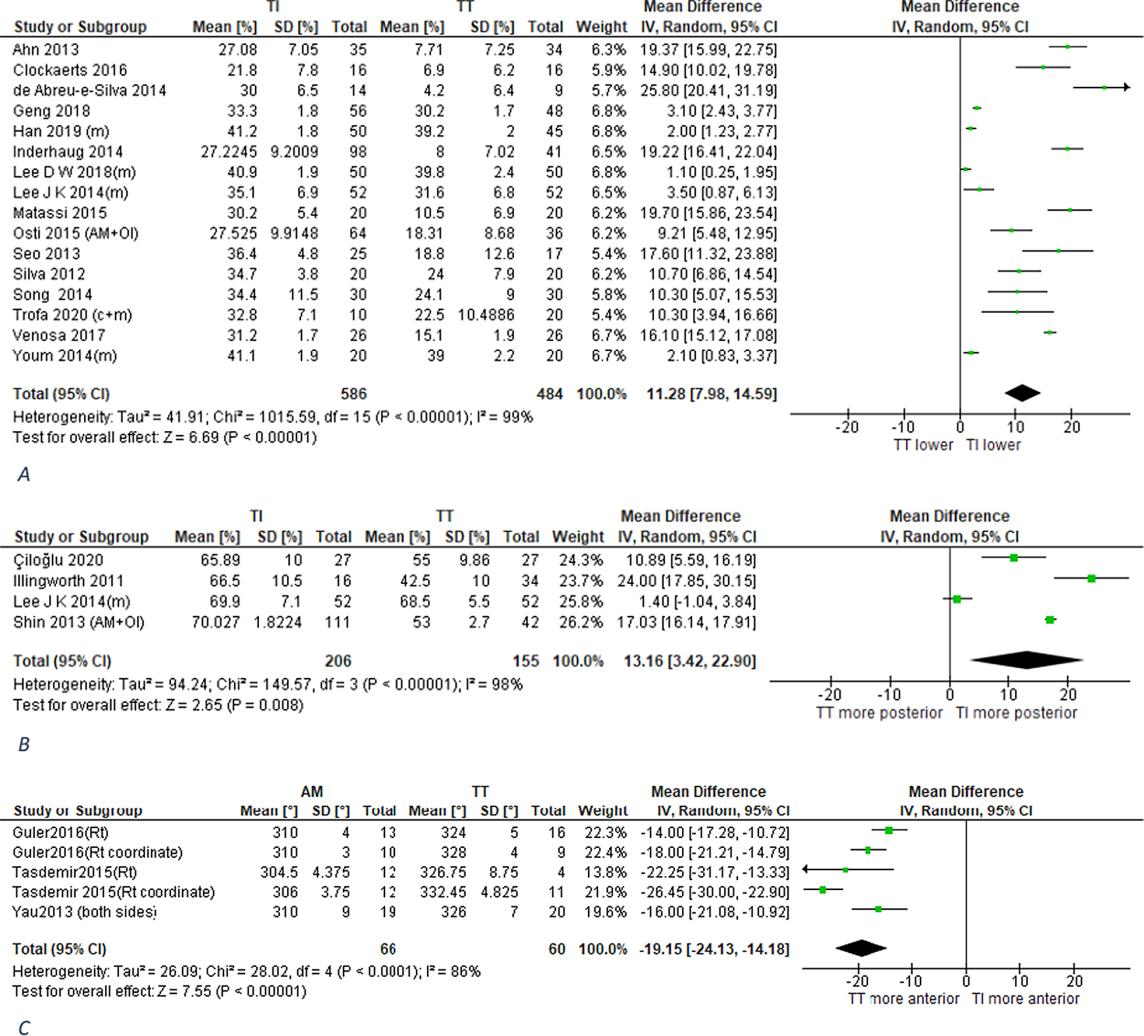
Meta-analysis of aperture coronal plane position. **A** Perpendicular to BL measured on 3DCT by quadrant method (Higher percentage is defined as lower aperture location). **B** Along AP axis measured on 3DCT by anatomic coordinate axis method (Higher percentage is defined as more posterior aperture location). **C** Along AP axis measured on axial MRI by clock face method (Higher degrees is defined as more anterior aperture position). (c + m): combined conventional and modified TT groups. (AM + OI): combined AM and OI groups

**Fig. 5:**
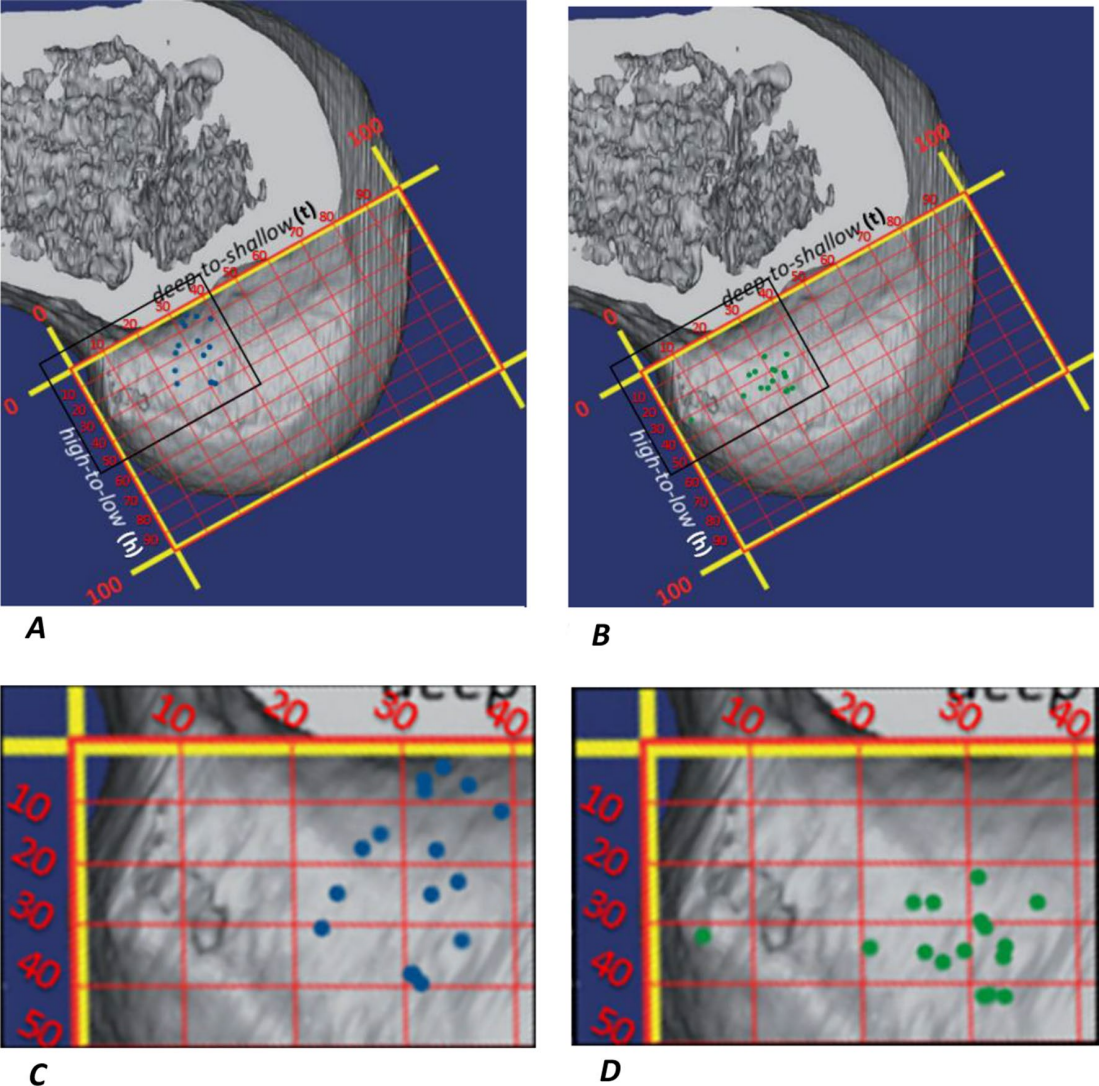
Femoral apertures placed by each technique [transtibial (**A**) and independent (**B**)] quantified by quadrant method on 3D CT reconstructed profile view of medial wall of lateral femoral condyle. The area in the black square frame contains 16 small points representing the centers of femoral apertures placed in 16 studies and synthesized in forest plots presented in Figs. 4A and 7A. This is magnified in **C** and **D**, respectively. h: line perpendicular to the Blumensaat’s line, t: line parallel to the Blumensaat’s line

In 16 studies (1070 patients), TI technique significantly lowered the position of the placed aperture than TT technique as measured by the quadrant method [71] on 3D CT (Figs. 4A, 5) [21, 22, 34, 41, 42]. A consistent finding was demonstrated in 2 studies (102 patients) [45, 56] as measured by quadrant method on radiography (Additional file 6) and in one study (105 participants) [19] as measured by method proposed by Heming [72] on tunnel AP radiograph. Also, on the same projection of distal femur (tunnel radiograph), Mirzatolooei [35] demonstrated a consistent finding using Sommer's method [73]

2. Along AP anatomical axis

I. As percentage ratio of an overall scaling dimension from LFC or intercondylar notch

**Fig. 6:**
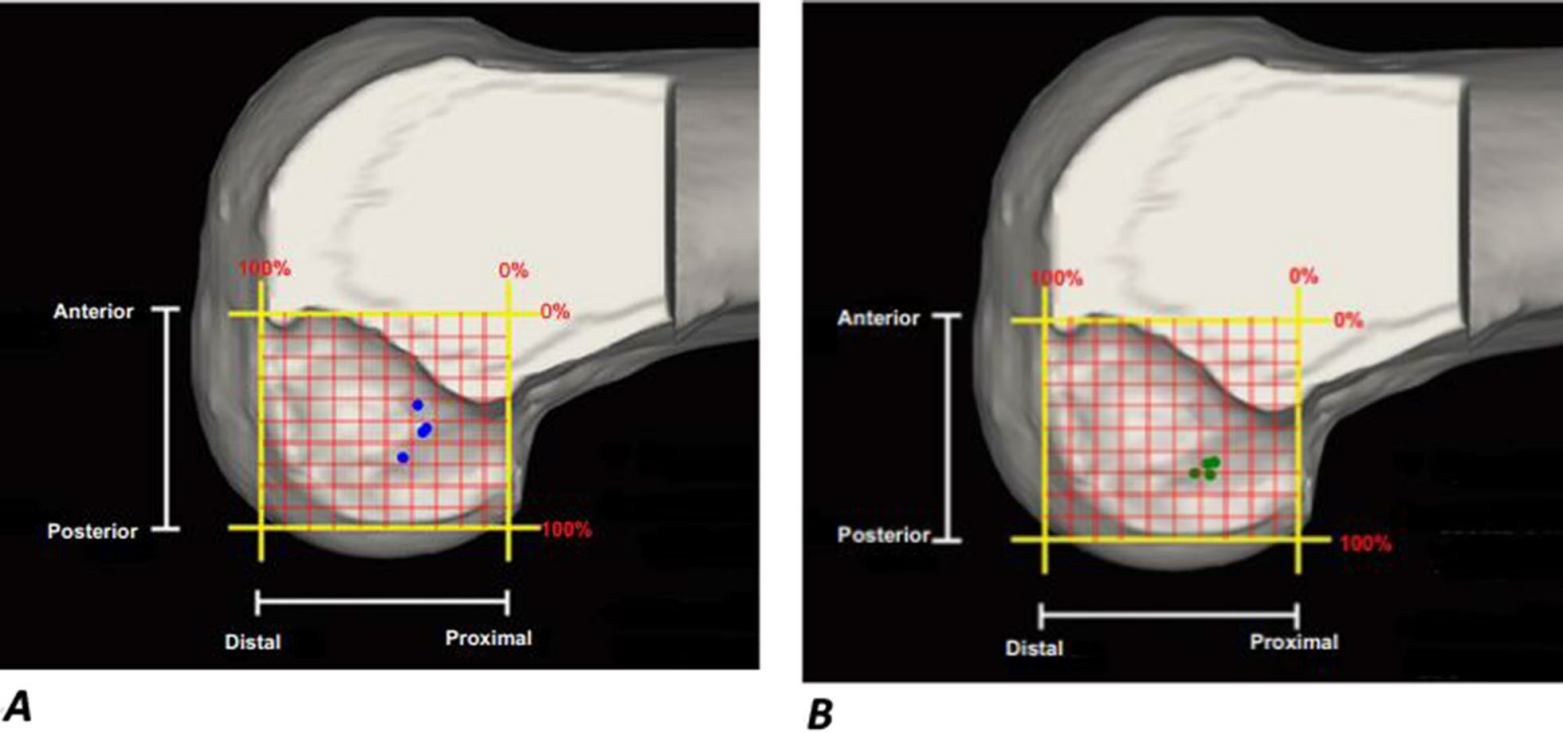
Femoral aperture placed by each technique [transtibial (**A**) and independent (**B**)] quantified by anatomic coordinate axis method on 3D CT reconstructed profile view of medial wall of lateral femoral condyle. Each figure includes 4 small points representing the centers of femoral apertures placed in 4 studies and synthesized in forest plots presented in Figs. 4B and 7B

In 4 studies (361 patients), TI technique placed femoral aperture in significantly more posterior position than TT technique as measured by ACA method [74] on 3D CT (Figs. 4B, 6) [29, 33, 40]. A consistent finding was demonstrated in 3 studies (126 participants) as measured by clock face method [75] on axial MRI (Fig. 4C) [25, 43, 47]. Also, in one study (20 specimens) [59], TI technique significantly placed femoral aperture significantly more posterior than TT technique as measured by method proposed by Heming on axial CT view. However, it may worth a little, to report that one study (20 specimens) [51] demonstrated that there is nonsignificant difference in aperture position along AP anatomical axis as measured by clock face method directly on specimens. However, this study is used an anterior target point at 11 o'clock position.

II. As distance in mm from a fixed anatomic landmark

A radiological study [36] using MRI performed on 61 participants demonstrated that the posterior margin of aperture placed by TI technique was significantly at more posterior distance from the over-the-top point (OTT) than TT technique. A consistent result was demonstrated on 20 specimens (one study) [60] where TI technique placed aperture center significantly closer to the inferior articular surface (IAS) than TT technique as measured on profile 3D CT view of medial wall of LFC. Contradictory to these findings, there was nonsignificant difference between both techniques in the distance of aperture to anterior notch tip as measured directly on 10 specimens (one study) [55]. Also, qualitative synthesis showed inconsistent results of 2 studies (40 specimens) assessing the distance of aperture inferior edge to inferior articular surface (IAS) on profile 3D CT view of medial wall of LFC. While one of them [63] showed nonsignificant difference between both techniques, the other [59] showed that TI technique placed aperture at significantly closer distance than TT technique.

B. Sagittal plane position

1. Along BL as percentage ratio of an overall scaling dimension from LFC or intercondylar notch

**Fig. 7:**
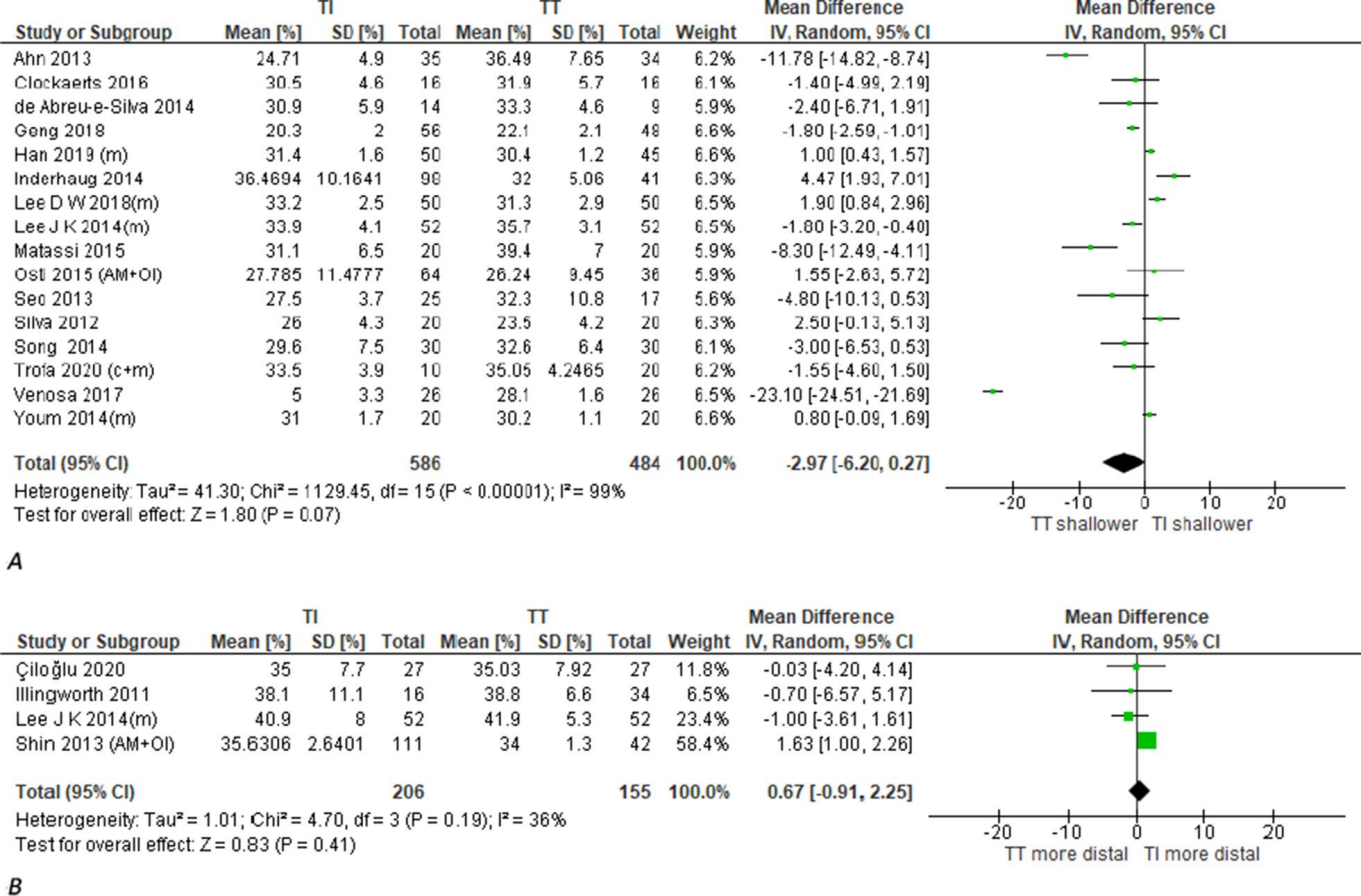
Meta-analysis of aperture sagittal plane position. **A** Along BL measured on 3DCT by quadrant method (Higher percentage is defined as shallower aperture position). **B** Along DP axis measured on 3DCT by anatomic coordinate axis method (Higher percentage is defined as more distal aperture position). (c + m): combined conventional and modified TT groups. (AM + OI): combined AM and OI groups

In 16 studies (1070 patients), there was nonsignificant difference between both techniques in the position of placed aperture along BL as measured by quadrant method [71] on 3D CT (Figs. 5, 7A) [21, 22, 34, 41, 42]. Using the same measurement method on radiography and MRI, a consistent result was demonstrated on 102 patients (2 studies) [45, 56] and 87 patients (2 studies) [25, 76], respectively (Additional file 6). Also, qualitative synthesis of results of 2 studies (80 patients) [13, 56] showed nonsignificant difference between both techniques in the position of anterior margin of placed aperture along BL as measured by Aglietti method [77] on radiography. Contradictory to the previous findings, TI technique placed femoral aperture at a significantly shallower position than the TT technique as measured by Harner method [77] on radiography in 2 studies (92 participants) [28, 46] (Additional file 6). A consistent finding was demonstrated in one study (30 patients) [20], where the placed femoral aperture screw head of TI technique was at a significantly shallower position than that of the TT technique as measured by the quadrant method on radiography. Also, in one study (12 specimens) [65], TI technique placed femoral aperture anterior margin at a significantly shallower position than the TT technique as measured by Aglietti method [77] directly on specimen.

2. Along PD anatomical axis

I. As percentage ratio of an overall scaling dimension from LFC or intercondylar notch

In 3 studies (361 patients) [25, 43, 47] there was nonsignificant difference between both techniques in the PD position of placed aperture as measured by the ACA method [74] on 3D CT (Figs. 6, 7B) [29, 33, 40]. Contradictory to that, in one study (100 patients) [37], TI technique placed femoral aperture at a significantly more distal position than the TT technique as measured by clock face method on CT coronal view.

II. As distance in mm from a fixed anatomic landmark

Nonsignificant difference was demonstrated in 3 studies, the first (20 specimens) [51] and second (20 specimens) [59] measured the distance of aperture posterior edge to posterior articular surface on specimen and on CT, respectively, and the third (61 participants) [36] measured the distance of aperture posterior edge to OTT point on MRI. Contradictory to that a significant difference between both techniques was demonstrated. The direction of that intervention effect was diverse among studies. In 2 studies (40 specimens) [54, 60], the aperture center placed by TI technique was closer to PAS than TT technique as measured on specimen and CT. Other 2 studies showed a more distal position of F aperture placed by TI technique with the first (10 specimens) [55] measuring the distance of aperture center to anterior notch tip and the second (20 specimens) [63] measuring distance of aperture anterior edge to anterior articular surface on profile 3D CT view of medial wall of LFC. For more detailed data, see summary of findings table of indirect methods (Table 2) [31].

**Table 2:** Summary of findings table of indirect outcomes

Outcome	Illustrative comparative risks		No of participants (studies)
	Assumed risk	Corresponding risk (95% CI)	
*Aperture coronal plane position as % of scaling dimension*			
a. Perpendicular to BL Quadrant method			
On CT On radiograph Heming method on tunnel radiograph Sommer method on tunnel radiograph	24% 16.3% 61.7° 0%, 17%, and 83% in Zone D, A, and B, respectively	11.3% lower position (8% lower: 14.6 lower) 4.45% lower position (1.9% lower to 7% lower) 5.8° lower (7.75° lower to 3.9° lower) 17%, 48%, and 34% in Zone D, A, and B, respectively	1070 (16 studies^a^) 102 (2 studies^b^) 105 (1 study) 105 (1 study)
b. Along AP axis Anatomic coordinate axis method on CT			
Clock face method On specimen On MRI Heming method on axial CT	56.2% 25.5° 327° 63.3°	13.6% more posterior (3.4% more posterior to 22.9% more posterior) 4.50° more anterior (3.15° more posterior to 12.15° more anterior) 19.15° more posterior (24.1° more posterior to 14.2° more posterior) 10.6° more posterior	359 (4 studies^c^) 20 (1 study) 126 (3 studies) 20 (1 study)
c. Mediolateral position%	NE	NE^d^	92 (2 studies)
*Aperture coronal plane position as distance from fixed anatomic landmark*			
*On specimen*			
Distance of aperture center to ANT along AP axis	19 mm posterior	0.6 mm more anterior (0.8 mm more posterior to 2 mm more anterior)	10 (1 study)
*On CT*			
Distance of aperture center to IAS Distance of aperture to IAS	11.8 mm NE	2.4 mm closer (3.6 mm closer to 1.8 mm closer) NE^e^	20 (1 study) 40 (2 studies)
*On MRI*			
Distance of aperture center to O-t-T along AP axis	1.4 mm posterior	7 mm more posterior (6.65 mm more posterior to 7.35 mm more posterior)	61 (1 study)
Aperture sagittal plane position as % of scaling dimension			
a. Along BL Quadrant method			
On CT On radiograph On MRI Harner method on radiograph Aglietti method On radiograph On specimen Aperture screw head position by quadrant method on radiograph	30.9% 32.2% 15.2% 26.6% NE 52.7% 25%	3% deeper (6.2% deeper to 0.3% shallower) 2.4% deeper (9.5% deeper to 4.7% shallower) 2.9% deeper (5.9% deeper to 0.1% shallower) 12.5% shallower (9.9% shallower:15% shallower) NE^f^ 8% shallower (4.2% shallower: 11.8% shallower) 5.7% shallower (2.7% shallower:8.7% shallower)	1070 (16 studies^a^) 102 (2 studies^b^) 87 (2 studies) 92 (2 studies) 80 (2 studies) 12 (1 study) 30 (1 study)
b. Along PD axis			
Anatomic coordinate axis method on CT Heming method on coronal CT	37.9% 74.67°	0.6% more distal (0.9% more proximal to 2.25% more distal) 7° more distal (9° more distal to 5° more distal)	359 (4 studies^c^) 100 (1 study)
*Aperture sagittal plane position as distance from fixed anatomic landmark*			
*On specimen*			
Distance of aperture center to ANT along PD axis Distance of aperture posterior edge to PAS Distance of aperture center to PAS	26.3 mm proximal 2.32 mm 6.1 mm	5.2 mm more distal (9.5 mm more distal to 0.9 mm more distal) 0.04 mm further (0.3 mm closer to 0.3 mm further) 0.85 mm closer (1 mm closer to 0.7 mm closer)	10 (1 study) 20 (1 study) 20 (1 study)
*On CT*			
Distance of aperture center to PAS Distance of aperture posterior edge to PAS Distance of aperture anterior edge to AAS	10.8 mm 3.36 mm 9.9 mm	3.9 mm closer (4.6 mm closer to 3.2 mm closer) 0.86 mm closer (NS) 4.9 mm closer (6.3 mm closer to 3.5 mm closer)	20 (1 study) 20 (1studiy) 20 (1 study)
*On MRI*			
Distance of aperture center to O-t-T along PD axis	8.6 mm distal	0.4 mm more distal (0.3 mm more proximal to 1.1 mm more distal)	61 (1 study)

Harner method: aperture position % from whole BL; Aglietti method: aperture anterior edge position; NE: not estimable; ANT: anterior notch tip; IAS: inferior articular surface; PAS: posterior articular surface; O-t-T: over-the-top point; NS: nonsignificant

^a^Other 2 studies [31, 66] whose results could not be pooled, measured the same outcome and showed inconsistent results

^b^A third study [23] whose results could not be pooled, measured the same outcome, and demonstrated a consistent result

^c^A fifth study [27] whose results could not be pooled, measured the same outcome, and demonstrated a consistent result

^d^Qualitative synthesis: The results were consistent. Both studies [28, 46] showed that TI technique placed F aperture at significantly more lateral position

^e^Qualitative synthesis: The results were inconsistent. Tompkins 2013 found nonsignificant difference and Larson found TI technique placed aperture significantly closer with mean difference of 6 mm

^f^Qualitative synthesis: The results were consistent. Both studies [13, 56] showed that there was nonsignificant difference between both techniques in position along BL

#### Comparison of tibial aperture position between TT and TI techniques

A. Tibial aperture anteroposterior (AP) position

In 13 studies (740 patients), TT technique placed tibial aperture in a significantly more posterior position than TI technique as measured on CT [MD: 2.64% more posterior (95% CI: 4.42–0.86%)] (Additional file 7). Also, in one study (30 patients) assessing the distance between tibial aperture center and footprint center in AP direction on MRI, TT technique placed tibial aperture at more posterior distance from footprint than TI technique with a mean difference of 6.5 mm [18]. In another study analyzing 20 specimens directly, TT technique placed significantly more apertures in the posterior third of same knee footprint than TI technique [62]. Contradictory to the pervious findings, there was nonsignificant difference between both techniques in the tibial aperture AP position measured on MRI and radiography in 4 studies (156 participants) and 2 studies (92 participants), respectively (Additional file 7). Another study whose results could not be pooled measured the tibial aperture anterior margin AP position on radiography and demonstrated a consistent result [13]. In addition, the difference in AP position%, measured on MRI, between tibial aperture and footprint of contralateral knee was nonsignificant between both techniques in one study (20 patients) [17].

B. Tibial aperture mediolateral (ML) position

There was nonsignificant difference between both techniques in the tibial aperture ML position measured on CT in 11 studies (703 participants) (Additional file 7). Using the same measurement method on radiography and MRI, a consistent result was demonstrated on 32 patients (one study) [46]; and 48 patients (one study) [25], respectively. In addition, there was nonsignificant difference between both techniques in recreating the ML footprint position of the contralateral knee in one study (30 participants) using MRI [18]. Also, the difference in ML position% measured on MRI between tibial aperture and footprint of contralateral knee was nonsignificant between both techniques in one study (20 patients) [17]. However, it may worth a little, to report that in one study (60 participants) TT technique placed tibial aperture in significantly more medial position than TI technique as measured on radiography [28].

### Subgroup analyses

While the type of TI technique (AM or OI) had nonsignificant effect on the intervention effect on aperture position perpendicular to BL measured on CT, the presence or absence of modifications in TT technique had significant effect on intervention effect on the same outcome. (Details are presented in Additional file 8). Because the data in the analyzed subgroups should be independent, both Osti et al. and Tofra et al. studies were excluded from the first and second subgroup analyses, respectively. Each of them has intervention group that will contribute to both analyzed subgroups. However, these excluded three-arm studies also emphasize the results of subgroup analyses. Osti et al. study demonstrated nonsignificant difference between AM and OI techniques in the height of placed aperture perpendicular to BL measured on CT. Torfa et al. study demonstrated that mTT technique significantly lowered the position of placed aperture than conventional TT technique.

### Sensitivity analysis

The intervention effect on aperture position perpendicular to BL measured on 3D CT differed a lot between the primary analysis (i.e., including all studies) and the sensitivity analysis in which we excluded observational studies. (Details are presented in Additional file 9).

## Discussion

The key findings of the present study indicated that there was nonsignificant difference between TI and TT techniques in the distance from femoral aperture center to footprint center. If there was a difference (in the minority of included studies), it was that the TI technique might place aperture center closer to footprint center than TT technique but with a small mean difference. Moreover, both techniques were unable to accurately recreate the anatomic footprint position. This could be explained by the inability to visualize the footprint in two thirds of included studies and the utilization of the general “rules of thumb” to provide an approximate location for aperture placement which are not applicable to all individuals. Findings also indicated the following.

Regarding aperture placement in the coronal plane, there might be a difference between both techniques in the ability to achieve footprint position along AP anatomical axis which was that TI technique might place the femoral aperture center at less anterior distance from same knee footprint center with a small mean difference. Also, TI technique placed aperture at significantly more posterior position than TT technique. In the direction perpendicular to BL, TI technique significantly lowered the position of the placed aperture than TT technique.

Regarding aperture placement in the sagittal plane, there was nonsignificant difference between both techniques in the ability to achieve footprint position along PD anatomical axis. If there was a difference, it was that the TI might place aperture center at less proximal distance from footprint center than TT technique with moderate mean difference. Also, there was nonsignificant difference between both techniques in the position of aperture placed along both BL and PD anatomical axis. If there was a difference, there was inconsistency in the direction of the intervention effect.

Regarding the effect of each technique on the tibial aperture position, there might be a difference between both techniques in tibial aperture AP position which was that TT technique might place the tibial aperture at more posterior position than TI technique. However, in 13 studies assessing tibial aperture AP position on CT, the difference looked clinically insignificant as both the point of estimate of the intervention effect (2.64%) measured on CT in 13 studies and its 95% confidence interval (4.42–0.86%) were less than the reported average anatomical range of tibial footprint center AP position (7%) [78].

*Relevance of our findings to the recent ACL direct/indirect insertion concept*: The central axis of ACL direct insertion (= parallel to intercondylar ridge (ICR)) is inclined 70° and 30° with the BL and PD anatomical axis, respectively [1, 66] (Fig. 2)*.* This could explain why the outcomes assessing aperture position along and perpendicular to BL, contradictory to those assessing it along AP and PD anatomical axes, could directly answer the question of the ability of each technique to properly position the aperture according to the new concept of direct and indirect fibers of ACL femoral insertion. As the aperture to be performed is smaller and geometrically distinct from the area occupied by ACL femoral insertion, researchers advised recently to place aperture eccentrically in the footprint where the structurally and functionally important direct fibers lie [1]. Putting altogether, we could suggest that the more anterior and distal placement along AP and PD anatomical axes would be more advantageous, not the reverse. Also, the differential in aperture placement along the perpendicular to BL, which is nearly parallel to ICR, may not be so crucial if the aperture is placed closely behind the ICR. However, the shallower placement along BL would be highly more advantageous. Fortunately, sagittal plane aperture position, whether along PD or along BL, is adjustable by changing offset guide size even in TT technique.

Regarding participants included in our review, in cadaveric studies, the average age of specimens was 67.5 years which was older than the average young age of population undergoing ACL reconstruction. However, whenever reported, specimens with degenerative changes were excluded. In clinical studies, the average age was 28.5 years which is the average age group undergoing ACL reconstruction. Regarding intervention integrity, the greatest single variable in a surgical comparison study is the surgeon, while same surgeon performing the procedures reduces operative variability; the difference in surgeons with variant experience level in our studies could make our findings generalizable to all surgeon groups.

Our review analyzed femoral aperture placement in TT technique with all its suggested modifications and the TI technique whether AM or OI. In addition, our review analyzed the effect of each technique on tibial aperture position which could also affect the ACL graft obliquity. Our review included 55 studies involving a total of 2401 knees and analyzing femoral aperture placement using both approaches of indirectly comparing the quantified aperture placed by each technique and directly comparing the ability of each technique to create the anatomic footprint position.

There was substantial heterogeneity in the intervention effect on aperture position along and perpendicular to BL on CT scan, this could be explained first by the statistically demonstrated effect of mTT technique (see subgroup analysis) which could also explain the inconsistency in the results of Larson et al. [59] and Tompkin et al. [63] studies where Larson et showed a significantly lower aperture placement by TI technique and Tompkin et al. showed nonsignificant difference between both techniques. Second, the included studies utilized different targets for femoral aperture placement which varied according to the chronological evolution of the appropriate femoral aperture position and the in-between studies variability. However, this variation was only inter-study variation not intra-study variation. Our review included studies from the nineties of the twentieth century when the target for aperture placement was at a point as close as possible to the over-the-top point [55], or just deep to AM bundle site [56], or at 11 O’clock position [13]. Then, there were studies from the first decade and the early second decade of the twenty-first century when aperture placement aimed at high deep part of footprint to mimic the AM bundle [16, 36, 40, 51, 54]. However, most of our studies used a target point at footprint center or its surrogate by other localization strategies. Third, regarding femoral aperture localization strategies, we found that, whenever reported, they varied in different studies. They included mainly clock face method, footprint remnants, and fixed anatomic landmarks. The clock face method [75] is confusing as reported by some surgeons. Neither the knee flexion angle in which the clock face was applied, nor the transverse reference axis of the clock face were specified in our studies, especially that there are different methods for clock face referencing in the literature [72, 79]. The footprint remnants method could be ineffective with the increase in time between injury and surgery which varied in our studies. The utilized anatomic landmarks varied in our studies. Localization strategies also included intraoperative assistance by fluoroscopy and navigation system in two [14, 37] and one studies [62], respectively. Lastly, the potentially different surgical technique, utilized among the included studies, may explain the high heterogeneity in the intervention effect on aperture position along and perpendicular to BL. First, the AM portal technique varied in our studies. Drilling the femoral tunnel through an accessory AM portal, while viewing through standard AM one, might allow better visualization and proper localization of femoral aperture at the best desired position. Second, the performance of notchplasty varied in our studies (performed in 9 studies). Technically, the TT technique requires an accurate notchplasty to visualize the femoral footprint. In contrast when performing AM portal reconstruction, the AM portal allows visualization of footprint without the need for notchplasty (97). Third, tibia tunnel intraarticular target point varied in our studies. Tibial tunnel aiming anteriorly at tibial footprint would direct the guide to a significantly shallower femoral aperture position. Fourth, tibial tunnel diameter varied in our studies. In the TT technique, increasing tibial tunnel diameter allows more maneuverability of the guide and adjusting femoral aperture to the best desired position [68]. Lastly, posterior wall thickness, permitted by offset guide, varied in our studies. This might lead to variability in the sagittal position of placed femoral aperture. This could also explain the inconsistency in the direction of intervention effect on aperture sagittal plane position between Gavriliidis et al. [54] and Miller et al. [60] studies on one side and Grondvedt et al. [55] and Tompkins et al. [63] studies on the other side. While the first 2 studies found that TI technique placed aperture more proximal than TT technique, the other two found that TI technique placed aperture more distal than TT technique. The size of utilized offset guide differed in these studies (6 mm and 7 mm in the first and second two studies, respectively).

There are some limitations in our review. Due to our thoughts that the anatomical positioning of femoral aperture is an anatomical outcome that could not be confounded too much by independent intraoperative and post-operative variables, we included RCTs and observational studies in our review. However, we assessed that decision by performing sensitivity analysis on aperture position perpendicular to BL measured on CT. In this sensitivity analysis, the intervention effect changed a lot. So, our results in that outcome (especially the intervention effect size) should be interpreted cautiously. In addition, as explained previously, alteration of tibial tunnel starting point was not included in defining mTT technique in our review. This surgical factor might influence aperture placement in our studies. For instance, the better placement of aperture center in Gadikota et al. [53] study more than Kaseta et al. [58] (1.5 mm versus 4.5 mm both anterior and proximal distance to same knee footprint center) could be explained by difference in tibial tunnel starting point which was Piasecki’s modified point [68] in Gadikota et al. study and Morgan conventional point [70] in Kaseta et al. study.

Two previous reviews compared femoral aperture location following TT and TI techniques. The first one [8] included 6 observational studies. Authors demonstrated a similar finding to ours; that TI technique placed femoral aperture in a lower position than TT technique. However, they compared the quantified femoral aperture location of each technique using the quadrant method and ACA method on 3D CT without taking into consideration the difference in axis direction between BL and anatomical coordinates. This drawback could explain the demonstrated inconsistency in the intervention effect on femoral aperture deep-to-shallow position between measurement using quadrant and ACA methods. Also, the review did not directly answer the question of how each technique was able to recreate the anatomic footprint. Moreover, the authors took an improper statistical approach of including all groups of three-arm studies in the meta-analysis resulting in counting the control group twice in the pooling analysis, giving those individuals twice their weight. The second one [9], included both observational studies and RCTs. Although the authors demonstrated that the TI technique placed femoral aperture significantly closer to the footprint center than the TT technique, the difference was small of 2.69 mm. Also, the authors pooled studies assessing femoral aperture position on different imaging modality or its surrogate. This is obviously an inaccurate approach as these modalities have different diagnostic accuracy. Moreover, they took the same improper statistical approach of including all groups of three-arm studies in the meta-analysis.

## Conclusion

### Implication for practice

This systematic review and meta-analysis of clinical and cadaveric studies demonstrated that both techniques were unable to accurately recreate the anatomic femoral footprint position. To recreate the anatomic footprint position, surgeons may consider the patient-specific locations of footprint. It also demonstrated that while the difference between both techniques was nonsignificant in aperture placement in the sagittal plane, it was significant in the coronal plane. This coronal plane placement could be either along the perpendicular to BL or along the AP anatomical axis. Along the perpendicular to BL, this statistically significant difference could be overcome by modifications to TT technique and may be non-crucial according to the recent concept of functional ACL reconstruction. Along the AP anatomical axis, the demonstrated direction of intervention effect, which was more posterior placement of femoral aperture by TI technique, could be, to some extent, in favor of the TT technique according to recent concept of functional ACL reconstruction. In addition, the review demonstrated that TT technique might place the tibial aperture at more posterior position than TI technique and the difference looked clinically insignificant.

### Implication for research

We advise conducting comparative studies using accurate and similar localization strategies for femoral aperture placement. Also, we advise the introduction of new assessment tools that reference the femoral aperture position to the axis of direct fibers insertion and the use of these tools in new studies comparing the ability of each technique to properly place femoral aperture.

## Supplementary Information

**Additional file 1**. Search strategy.

**Additional file 2**. Characteristics of included studies table.

**Additional file 3**. Studies with modifications in TT technique.

**Additional file 4**. Femoral aperture localization strategy utilized in TT and TI technique in included studies.

**Additional file 5**. Metanalyses of direct outcomes.

**Additional file 6**. Metanalyses of indirect outcomes.

**Additional file 7**. Metanalyses of tibial aperture position.

**Additional file 8**. Subgroup analyses by variation in TI techniques {AM vs OI} (Fig. A) and by modifications to TT technique {conventional TT vs modified TT (mTT)} (Fig. B).

**Additional file 9**. Sensitivity analysis excluding low quality-design studies (observational studies).

## Data Availability

The datasets used and/or analyzed during the current study are available from the corresponding author on reasonable request.
