# Supplementary material for: Transtibial versus independent femoral tunnel drilling techniques for anterior cruciate ligament reconstruction: evaluation of femoral aperture positioning
Source: J Orthop Surg Res. 2022 Mar 18;17:166. doi: 10.1186/s13018-022-03040-5 (PMC8931956; doi:10.1186/s13018-022-03040-5)
Supplement: Supplementary file 1 — Additional file 1. Search strategy. [file 13018_2022_3040_MOESM1_ESM.docx]

Article title: Transtibial versus Independent Femoral Tunnel Drilling Techniques for Anterior Cruciate Ligament reconstruction: Evaluation of Femoral Aperture Positioning. A Systematic review and Meta-analysis

Journal name: Journal of Orthopaedic Surgery and Research

Author names and affiliation: Haitham K. Haroun^1^, Maged M. Abouelsoud^1^, Mohamed R. Allam ^2^, and Mahmoud M. Abdelwahab^1^

^1^ Orthopedic Department, Faculty of Medicine, Ain Shams University, Cairo, Egypt

^2^El Demerdash Hospital, Ain-Shams University, Cairo, Egypt

e-mail address of the corresponding author: haroun.haitham@med.asu.edu.eg

**Additional file 1: Search strategy**

MEDLINE **PubMed Platfrom**

Search number,Query,Sort By,Filters,Search Details,Results,Time

20,#3 AND #19,,from 1000/1/1 - 2021/3/1,"(((""anterior cruciate ligament""[MeSH Terms] AND 1000/01/01:2021/03/01[Date - Publication]) OR (""ACL""[Title/Abstract] AND 1000/01/01:2021/03/01[Date - Publication])) AND 1000/01/01:2021/03/01[Date - Publication] AND (((""tunnel position*""[Title/Abstract] AND 1000/01/01:2021/03/01[Date - Publication]) OR (""femoral aperture""[Title/Abstract] AND 1000/01/01:2021/03/01[Date - Publication]) OR (""femoral tunnel""[Title/Abstract] AND 1000/01/01:2021/03/01[Date - Publication]) OR (""femoral footprint""[Title/Abstract] AND 1000/01/01:2021/03/01[Date - Publication]) OR (""drilling technique""[Title/Abstract] AND 1000/01/01:2021/03/01[Date - Publication]) OR (""reaming technique""[Title/Abstract] AND 1000/01/01:2021/03/01[Date - Publication]) OR (""transtibial""[Title/Abstract] AND 1000/01/01:2021/03/01[Date - Publication]) OR (""anteromedial""[Title/Abstract] AND 1000/01/01:2021/03/01[Date - Publication]) OR (""medial portal technique""[Title/Abstract] AND 1000/01/01:2021/03/01[Date - Publication]) OR (""tibial tunnel independent technique""[Title/Abstract] AND 1000/01/01:2021/03/01[Date - Publication]) OR (""tibial independent""[Title/Abstract] AND 1000/01/01:2021/03/01[Date - Publication]) OR (""anatomic*""[Title/Abstract] AND 1000/01/01:2021/03/01[Date - Publication]) OR (""nonanatomic*""[Title/Abstract] AND 1000/01/01:2021/03/01[Date - Publication]) OR (""transportal""[Title/Abstract] AND 1000/01/01:2021/03/01[Date - Publication]) OR (""outside-in""[Title/Abstract] AND 1000/01/01:2021/03/01[Date - Publication])) AND 1000/01/01:2021/03/01[Date - Publication])) AND (1000/1/1:2021/3/1[pdat])","3,302",00:32:04

19,#4 OR #5 OR #6 OR #7 OR #8 OR #9 OR #10 OR #11 OR #12 OR #13 OR #14 OR #15 OR #16 OR #17 OR #18,,from 1000/1/1 - 2021/3/1,"((""tunnel position*""[Title/Abstract] AND 1000/01/01:2021/03/01[Date - Publication]) OR (""femoral aperture""[Title/Abstract] AND 1000/01/01:2021/03/01[Date - Publication]) OR (""femoral tunnel""[Title/Abstract] AND 1000/01/01:2021/03/01[Date - Publication]) OR (""femoral footprint""[Title/Abstract] AND 1000/01/01:2021/03/01[Date - Publication]) OR (""drilling technique""[Title/Abstract] AND 1000/01/01:2021/03/01[Date - Publication]) OR (""reaming technique""[Title/Abstract] AND 1000/01/01:2021/03/01[Date - Publication]) OR (""transtibial""[Title/Abstract] AND 1000/01/01:2021/03/01[Date - Publication]) OR (""anteromedial""[Title/Abstract] AND 1000/01/01:2021/03/01[Date - Publication]) OR (""medial portal technique""[Title/Abstract] AND 1000/01/01:2021/03/01[Date - Publication]) OR (""tibial tunnel independent technique""[Title/Abstract] AND 1000/01/01:2021/03/01[Date - Publication]) OR (""tibial independent""[Title/Abstract] AND 1000/01/01:2021/03/01[Date - Publication]) OR (""anatomic*""[Title/Abstract] AND 1000/01/01:2021/03/01[Date - Publication]) OR (""nonanatomic*""[Title/Abstract] AND 1000/01/01:2021/03/01[Date - Publication]) OR (""transportal""[Title/Abstract] AND 1000/01/01:2021/03/01[Date - Publication]) OR (""outside-in""[Title/Abstract] AND 1000/01/01:2021/03/01[Date - Publication])) AND (1000/1/1:2021/3/1[pdat])","269,847",00:31:23

18,outside-in[Title/Abstract],,from 1000/1/1 - 2021/3/1,"(""outside-in""[Title/Abstract]) AND (1000/1/1:2021/3/1[pdat])","2,317",00:30:25

17,transportal[Title/Abstract],,from 1000/1/1 - 2021/3/1,"(""transportal""[Title/Abstract]) AND (1000/1/1:2021/3/1[pdat])",175,00:29:18

16,nonanatomic*[Title/Abstract],,from 1000/1/1 - 2021/3/1,"(""nonanatomic*""[Title/Abstract]) AND (1000/1/1:2021/3/1[pdat])","1,492",00:28:37

15,anatomic*[Title/Abstract],,from 1000/1/1 - 2021/3/1,"(""anatomic*""[Title/Abstract]) AND (1000/1/1:2021/3/1[pdat])","261,448",00:27:47

14,tibial independent[Title/Abstract],,from 1000/1/1 - 2021/3/1,"(""tibial independent""[Title/Abstract]) AND (1000/1/1:2021/3/1[pdat])",2,00:27:13

13,tibial tunnel independent technique[Title/Abstract],,from 1000/1/1 - 2021/3/1,"(""tibial tunnel independent technique""[Title/Abstract]) AND (1000/1/1:2021/3/1[pdat])",5,00:26:19

12,medial portal technique[Title/Abstract],,from 1000/1/1 - 2021/3/1,"(""medial portal technique""[Title/Abstract]) AND (1000/1/1:2021/3/1[pdat])",23,00:25:43

11,anteromedial[Title/Abstract],,from 1000/1/1 - 2021/3/1,"(""anteromedial""[Title/Abstract]) AND (1000/1/1:2021/3/1[pdat])","4,177",00:24:49

10,transtibial[Title/Abstract],,from 1000/1/1 - 2021/3/1,"(""transtibial""[Title/Abstract]) AND (1000/1/1:2021/3/1[pdat])","2,329",00:22:06

9,reaming technique[Title/Abstract],,from 1000/1/1 - 2021/3/1,"(""reaming technique""[Title/Abstract]) AND (1000/1/1:2021/3/1[pdat])",43,00:21:29

8,drilling technique[Title/Abstract],,from 1000/1/1 - 2021/3/1,"(""drilling technique""[Title/Abstract]) AND (1000/1/1:2021/3/1[pdat])",252,00:20:42

7,femoral footprint[Title/Abstract],,from 1000/1/1 - 2021/3/1,"(""femoral footprint""[Title/Abstract]) AND (1000/1/1:2021/3/1[pdat])",135,00:19:00

6,femoral tunnel[Title/Abstract],,from 1000/1/1 - 2021/3/1,"(""femoral tunnel""[Title/Abstract]) AND (1000/1/1:2021/3/1[pdat])","1,337",00:18:14

5,femoral aperture[Title/Abstract],,from 1000/1/1 - 2021/3/1,"(""femoral aperture""[Title/Abstract]) AND (1000/1/1:2021/3/1[pdat])",13,00:17:40

4,tunnel position*[Title/Abstract],,from 1000/1/1 - 2021/3/1,"(""tunnel position*""[Title/Abstract]) AND (1000/1/1:2021/3/1[pdat])",546,00:16:51

3,#1 OR #2,,from 1000/1/1 - 2021/3/1,"((""anterior cruciate ligament""[MeSH Terms] AND 1000/01/01:2021/03/01[Date - Publication]) OR (""ACL""[Title/Abstract] AND 1000/01/01:2021/03/01[Date - Publication])) AND (1000/1/1:2021/3/1[pdat])","22,193",00:14:11

2,ACL[Title/Abstract],,from 1000/1/1 - 2021/3/1,"(""ACL""[Title/Abstract]) AND (1000/1/1:2021/3/1[pdat])","17,538",00:13:56

1,anterior cruciate ligament[MeSH Terms],,from 1000/1/1 - 2021/3/1,"(""anterior cruciate ligament""[MeSH Terms]) AND (1000/1/1:2021/3/1[pdat])","11,363",00:13:33

**CENTRAL**

Last Saved: 3/3/2021 13:30:23

Comment:

ID Search

#1 MeSH descriptor: [Anterior Cruciate Ligament] explode all trees

#2 (ACL):ti,ab,kw (Word variations have been searched) with Publication Year from 1990 to 2021, with Cochrane Library publication date Between Jan 1990 and Mar 2021, in Trials

#3 #1 OR #2 with Publication Year from 1990 to 2021, with Cochrane Library publication date Between Jan 1990 and Mar 2021, in Trials

#4 (tunnel position):ti,ab,kw with Publication Year from 1990 to 2021, with Cochrane Library publication date Between Jan 1990 and Mar 2021, in Trials

#5 (femoral aperture):ti,ab,kw with Publication Year from 1990 to 2021, with Cochrane Library publication date Between Jan 1990 and Mar 2021, in Trials

#6 (femoral tunnel):ti,ab,kw

#7 (femoral footprint):ti,ab,kw

#8 (drilling technique):ti,ab,kw

#9 (reaming technique):ti,ab,kw

#10 (transtibial):ti,ab,kw

#11 (nonanatom*):ti,ab,kw

#12 (tibial dependent):ti,ab,kw

#13 (anteromedial):ti,ab,kw

#14 (medial portal technique):ti,ab,kw

#15 (tibial tunnel-independent):ti,ab,kw

#16 (tibial independent):ti,ab,kw

#17 (anatom*):ti,ab,kw

#18 (transportal):ti,ab,kw

#19 (outside-in):ti,ab,kw

#20 #4 OR #5 OR #6 OR #7 OR #8 OR #9 OR #10 OR #11 OR #12 OR #13 OR #14 OR #15 OR #16 OR #17 OR #18 OR #19 with Publication Year from 1990 to 2021, with Cochrane Library publication date Between Jan 1990 and Mar 2021, in Trials

#21 #3 AND #20 with Publication Year from 1990 to 2021, with Cochrane Library publication date Between Jan 1990 and Mar 2021, in Trials

**Web of Science**

ID Search

#1 TS=(“Anterior Cruciate Ligament”)

#2 TS=(“ACL”)

#3 #1 or #2

#4 TS=("tunnel position")

#5 TS=("femoral aperture")

#6 TS=("femoral tunnel")

#7 TS=("femoral footprint")

#8 TS=("drilling technique")

#9 TS=("reaming technique")

#10 TS=(transtibial)

#11 TS=(nonanatom*)

#12 TS=("tibial dependent")

#13 TS=(anteromedial)

#14 TS=("medial portal technique")

#15 TS=("tibial tunnel-independent")

#16 TS=("tibial independent")

#17 TS=(anatom*)

#18 TS=(transportal)

#19 TS=(outside-in)

#20 #4 or #5 or #6 or #7 or #8 or #9 or #10 or #11 or #12 or #13 or #14 or #15 or #16 or #17 or #18 or #19

#21 #3 and #20
