# Supplementary material for: Transtibial versus independent femoral tunnel drilling techniques for anterior cruciate ligament reconstruction: evaluation of femoral aperture positioning
Source: J Orthop Surg Res. 2022 Mar 18;17:166. doi: 10.1186/s13018-022-03040-5 (PMC8931956; doi:10.1186/s13018-022-03040-5)
Supplement: Supplementary file 2 — Additional file 2. Characteristics of included studies table. [file 13018_2022_3040_MOESM2_ESM.docx]

Article title: Transtibial versus Independent Femoral Tunnel Drilling Techniques for Anterior Cruciate Ligament reconstruction: Evaluation of Femoral Aperture Positioning. A Systematic review and Meta-analysis.

Journal name: Journal of Orthopaedic Surgery and Research

Author names and affiliation: Haitham K. Haroun^1^, Maged M. Abouelsoud^1^, Mohamed R. Allam ^2^, and Mahmoud M. Abdelwahab^1^

^1^ Orthopedic Department, Faculty of Medicine, Ain Shams University, Cairo, Egypt

^2^El Demerdash Hospital, Ain-Shams University, Cairo, Egypt

e-mail address of the corresponding author: haroun.haitham@med.asu.edu.eg

**Additional file 2:** **Characteristics of included studies Table**

| **No** | **Study ID** | **Year** | **Research type** | **Study design** | **Study groups** | **Number of knees assessed** | **Imaging Modality (or its surrogate)** | **Minors score (24 max)** |
| --- | --- | --- | --- | --- | --- | --- | --- | --- |
| 1 | Abebe et al | 2009 | Clinical | Retrospective Cohort | TT vs OI | 16 (8, 8) | MRI | 17 |
| 2 | Aglietti et al | 1995 | Clinical | Retrospective cohort | TT vs OI | 50 (25, 25) | Radiograph | 17 |
| 3 | Ahn et al | 2013 | Clinical | Retrospective cohort | TT vs OI | 69 (34, 35) | CT | 16 |
| 4 | Albuquerque et al | 2007 | Basic science | Controlled trial | TT vs AM | 20 (10, 10) | Direct measurement on specimen | 17 |
| 5 | Arno et al | 2016 | Clinical | Retrospective cohort | TT vs AM | 20 (10, 10) | MRI | 17 |
| 6 | Bedi et al | 2011 | Basic science | Controlled trial | mTT vs AM | 10 (5, 5) | Direct measurement on specimen | 19 |
| 7 | Bowers et al | 2011 | Clinical | Retrospective cohort | TT vs AM | 30 (15, 15) | MRI | 18 |
| 8 | Chang et al | 2013 | Clinical | Retrospective cohort | TT vs AM | 105 (55, 50) | Radiograph | 17 |
| 9 | Cho et al | 2012 | Clinical | Retrospective cohort | TT vs AM | 30 (15, 15) | Radiograph | 19 |
| 10 | Çiloğlu et al | 2020 | Clinical | Prospective cohort | TT vs AM | 54 (27, 27) | CT | 15 |
| 11 | Clockaert et al | 2016 | Clinical | Prospective cohort | TT vs AM | 32(16, 16) | CT | 19 |
| 12 | De Abreu Silva et al | 2014 | Clinical | Retrospective cohort | TT vs AM | 23 (9, 14) | CT | 18 |
| 13 | Franceschi et al | 2013 | Clinical | Retrospective cohort | TT vs AM | 88 (46, 42) | Radiograph | 16 |
| 14 | Gadikota et al | 2012 | Basic science | RCT | TT vs AM vs OI | 8 (8, 8, 8) | Digitized 3D model | 19 |
| 15 | Gavriilidis et al | 2008 | Basic dcience | Controlled trial | TT vs AM | 10(10, 10) | Direct on specimen and photographed arthroscopic image | 17 |
| 16 | Geng et al | 2018 | Clinical | (quasi) RCT | TT vs AM | 104(48, 56) | CT | 23 |
| 17 | Giron et al | 1999 | Basic science | Controlled trial | TT vs AM vs OI | 30(10,10 ,10) | Radiograph | 20 |
| 18 | Grontvedt et al | 1996 | Basic science | Controlled trial | TT vs OI | 10 (5, 5) | Digitized 3D model | 17 |
| 19 | Guler et al | 2016 | Clinical | Retrospective cohort | TT vs AM | 48 (25, 23) | MRI | 17 |
| 20 | Han et al | 2019 | Clinical | RCT | mTT vs AM | 95 (45, 50) | CT | 18 |
| 21 | Harner et, al | 1994 | Clinical | Prospective cohort | TT vs OI | 50 (26, 24) | Radiograph | 13 |
| 22 | Hart et al | 2018 | Clinical | Prospective cohort | TT* vs AM^*^ | 41 (20, 21) | MRI (mapped on scaled schematic diagram) | 16 |
| 23 | Hensler et al | 2013 | Clinical | Retrospective cohort | TT vs AM | 47 (27, 20) | CT | 16 |
| 24 | Hussin et al | 2018 | Clinical | RCT | mTT vs AM | 60 (30, 30) | Radiograph | 15 |
| 25 | Illingworth  et al | 2011 | Clinical | Retrospective cohort | TT vs TI | 50 (34, 16) | CT | 18 |
| 26 | Inderhaug et al | 2016 | Clinical | Prospective cohort | TT vs AM* | 139 (41, 98) | CT | 18 |
| 27 | Jaecker et al | 2017 | Clinical | Retrospective cohort | TT vs AM | 101 (64, 37) | CT | 16 |
| 28 | Jennings et al | 2017 | Basic science | RCT | TT vs mTT (hybrid) vs AM | 72 (16, 28, 28) | Digitized 3D model | 17 |
| 29 | Kaseta et al | 2008 | Basic science | Controlled trial | TT vs OI | 12(12, 12) | Digitized 3D model | 22 |
| 30 | Larson et al | 2012 | Basic science | Controlled trial | TT vs AM* vs OI | 20 (5,10, 5) | CT | 17 |
| 31 | Lee D.W.  et al | 2018 | Clinical | Retrospective cohort | mTT vs OI | 100 (50, 50) | CT | 21 |
| 32 | Lee J. K, et al | 2014 | Clinical | Retrospective cohort | mTT vs AM | 104 (52, 52) | CT | 21 |
| 33 | Matassi et al | 2015 | Clinical | Prospective cohort | TT vs OI | 40 (20, 20) | CT | 19 |
| 34 | Miller et al | 2011 | Basic science | RCT | TT vs AM | 20 (10, 10) | CT | 21 |
| 35 | Mirzatolooei et al, | 2012 | Clinical | Prospective cohort | TT vs AM | 105 (47, 58) | Radiograph | 15 |
| 36 | Noh et al, | 2013 | Clinical | RCT | TT vs AM | 61 (30, 31) | MRI | 22 |
| 37 | Osti et al, | 2015 | Clinical | Retrospective cohort | TT vs AM vs OI | 100 (36, 32, 32) | CT | 19 |
| 38 | Pascual et al, | 2013 | Clinical | Retrospective cohort | TT vs AM | 40 (17, 23) | Radiograph | 16 |
| 39 | Robert et al, | 2013 | Basic science | RCT | TT vs AM vs OI | 13 (13, 13, 13) | CT | 19 |
| 40 | Seo et al | 2013 | Clinical | Retrospective cohort | TT vs OI | 42 (17, 25) | CT | 15 |
| 41 | Shin et al, | 2013 | Clinical | Retrospective cohort | TT vs AM vs OI | 153 (42,73, 38) | CT | 19 |
| 42 | Silva et al, | 2012 | Clinical | Prospective cohort | TT vs AM | 40 (20, 20) | CT | 18 |
| 43 | Song et al, | 2014 | Clinical | Prospective cohort | TT vs AM | 60 (30, 30) | CT | 22 |
| 44 | Steiner et al, | 2009 | Basic science | Quasi (RCT) | TT vs AM | 20 (10, 10) | Direct measurement on specimen | 17 |
| 45 | Tasdemir et al, | 2015 | Clinical | Retrospective cohort | TT vs AM | 39 (15, 24) | MRI | 19 |
| 46 | Trofa et al, | 2020 | Clinical | RCT | TT vs mTT vs AM | 30 (10, 10, 10) | CT | 24 |
| 47 | Tompkis2012 et al | 2012 | Basic science | Controlled trial | mTT vs AM | 20 (10, 10) | CT | 19 |
| 48 | Tompkins2013 et al | 2013 | Basic science | Controlled trial | mTT vs AM | 20 (10, 10) | CT | 18 |
| 49 | Tudsico et al | 2012 | Basic science | Controlled trial | TT vs AM | 12 (6, 6) | Direct measurement on specimen | 20 |
| 50 | Venosa et al, | 2017 | Clinical | RCT | TT vs AM | 52 (26, 26) | CT | 20 |
| 51 | Wolf et al, | 2014 | Basic science | Controlled trial | TT vs AM vs OI | 67 (23, 22, 22) | CT | 18 |
| 52 | Xu eta al, | 2011 | Clinical | Retrospective cohort | TT vs AM | 72 (53, 19) | Radiograph | 16 |
| 53 | Yanasse et al, | 2016 | Clinical | Prospective cohort | TT vs OI | 32 (14, 18) | Radiograph | 18 |
| 54 | Yau et al, | 2013 | Clinical | Prospective cohort | mTT vs AM | 39 (20, 19) | MRI | 20 |
| 55 | Youm et al, | 2014 | Clinical | RCT | mTT vs AM | 40 (20, 20) | CT | 20 |

^*^ Intervention group divided into 2 subgroups.
