# Supplementary material for: Transtibial versus independent femoral tunnel drilling techniques for anterior cruciate ligament reconstruction: evaluation of femoral aperture positioning
Source: J Orthop Surg Res. 2022 Mar 18;17:166. doi: 10.1186/s13018-022-03040-5 (PMC8931956; doi:10.1186/s13018-022-03040-5)
Supplement: Supplementary file 3 — Additional file 3. Studies with modifications in TT technique. [file 13018_2022_3040_MOESM3_ESM.docx]

Article title: Transtibial versus Independent Femoral Tunnel Drilling Techniques for Anterior Cruciate Ligament reconstruction: Evaluation of Femoral Aperture Positioning. A Systematic review and Meta-analysis

Journal name: Journal of Orthopaedic Surgery and Research

Author names and affiliation: Haitham K. Haroun^1^, Maged M. Abouelsoud^1^, Mohamed R. Allam ^2^, and Mahmoud M. Abdelwahab^1^

^1^ Orthopedic Department, Faculty of Medicine, Ain Shams University, Cairo, Egypt

^2^El Demerdash Hospital, Ain-Shams University, Cairo, Egypt

e-mail address of the corresponding author: haroun.haitham@med.asu.edu.eg

**Additional file 3: Studies with modifications in TT technique**

| No | Study | Modification technique |
| --- | --- | --- |
| 1 | **Lee JK et al, 2014** | Anterior drawer, Varus force, and External rotation to the tibia. |
| 2 | **Youm et al, 2014** | Varus and internal rotation of the tibia. |
| 3 | **Han et al, 2019** | Varus and internal rotation to the tibia. |
| 4 | **Hussin et al, 2013** | Posterolateral over the top notchplasty.  Tibial aperture beveling. |
| 5 | **Yau et al, 2013** | Posterolateral notchplasty. |
| 6 | **Jenning et al, 2017** | TT flexible wire was directed by a novel offset femoral drill guide inserted through AM portal. |
| 7 | **Trofa et al, 2020** | TT flexible wire was directed by a novel offset femoral drill guide inserted through AM portal |
| 8 | **Lee DW et al, 2018** | Trough performed through AM portal was used to force the TT flexible guide wire to predetermined anatomical position. |
| 9 | **Tompkins et al, 2012** | Tibial aperture beveling. |
| 10 | **Tompkins et al,2013** | Tibial aperture beveling. Varus stress to the tibia. |
| 11 | **Bedi at al, 2011** | Flexion and rotation of the tibia were adjusted to allow for best possible approximation of footprint center. |
