# Supplementary material for: Transtibial versus independent femoral tunnel drilling techniques for anterior cruciate ligament reconstruction: evaluation of femoral aperture positioning
Source: J Orthop Surg Res. 2022 Mar 18;17:166. doi: 10.1186/s13018-022-03040-5 (PMC8931956; doi:10.1186/s13018-022-03040-5)
Supplement: Supplementary file 4 — Additional file 4. Femoral aperture localization strategy utilized in TT and TI technique in included studies. [file 13018_2022_3040_MOESM4_ESM.docx]

Article title: Transtibial versus Independent Femoral Tunnel Drilling Techniques for Anterior Cruciate Ligament reconstruction: Evaluation of Femoral Aperture Positioning. A Systematic review and Meta-analysis

Journal name: Journal of Orthopaedic Surgery and Research

Author names and affiliation: Haitham K. Haroun^1^, Maged M. Abouelsoud^1^, Mohamed R. Allam ^2^, and Mahmoud M. Abdelwahab^1^

^1^ Orthopedic Department, Faculty of Medicine, Ain Shams University, Cairo, Egypt

^2^El Demerdash Hospital, Ain-Shams University, Cairo, Egypt

e-mail address of the corresponding author: haroun.haitham@med.asu.edu.eg

**Additional file 4-a: Femoral aperture localization strategy utilized in TT technique in included studies.**

| Localization strategy | | | No of studies | |
| --- | --- | --- | --- | --- |
| Using  offset guide  (OG) | OG only | | **12** | |
|  | OG and another method | | **25** | |
|  | +FP | | 12 | |
|  | +CF | | 6 | |
|  | +FP + CF | | 1 | |
|  | + FP+LM | | 1 | |
|  | +Fluoroscopy | | 1 | |
|  | +FP + Fluoroscopy | | 1 | |
|  | + LM | | 2 | |
|  | +CF + LM | | 1 | |
| Free hand | | LM | 1 | **9** |
|  |  | CF | 2 |  |
|  |  | FP | 2 |  |
|  |  | LM+CF | 3 |  |
|  |  | LM+FP | 1 |  |

FP: footprint; all at center except one study deep to AMB (Giron et al).

LM: Landmarks; included (posterior notch- posterior articular surface- inferior articular surface- posterior wall and notch roof- lateral intercondylar ridge and bifurcate ridge- over the top point).

CF: clock face; with time at (10, 10:30 to 11, and 11 o’clock)

**Additional file 4-b: Femoral aperture localization strategy utilized in TI technique in included studies.**

| Localization strategy | | No of studies | |
| --- | --- | --- | --- |
| Using FP^*^ | FP only | 18 | |
|  | FP and another method | 17 | |
|  | +FP+ OG | 4 | |
|  | +FP+ OG + LM | 3 | |
|  | +FP+ Fluoroscopy | 1 | |
|  | +FP+ OG +Fluoroscopy | 1 | |
|  | +FP + LM | 7 | |
|  | +FP+LM+CF | 1 | |
| Not using FP | OG + CF | 5 | 16 |
|  | OG+LM | 2 |  |
|  | OG | 1 |  |
|  | LM+CF | 2 |  |
|  | CF only | 1 |  |
|  | LM only | 2 |  |
|  | OG + CF + LM | 1 |  |
|  | OG+ Fluoroscopy | 1 |  |
|  | Surgical navigation system | 1 |  |

FP: footprint; **^*^** always at the FP center except in four studies (just deep to AM bundle in Giron et al, shallow to AM bundle in Gavriilidis et al, at AM bundle in Noh et al and Shin et al studies).

LM: Landmarks; included (over the top point- posterior and inferior articular surfaces- lateral intercondylar and bifurcate ridges- apex of deep cartilage in Hart et al- posterior synovial fold in Clockaerts et al study).

CF: clock face; with time at (10, 10 to10:30, and 11 o'clock)
