# Supplementary material for: Transtibial versus independent femoral tunnel drilling techniques for anterior cruciate ligament reconstruction: evaluation of femoral aperture positioning
Source: J Orthop Surg Res. 2022 Mar 18;17:166. doi: 10.1186/s13018-022-03040-5 (PMC8931956; doi:10.1186/s13018-022-03040-5)
Supplement: Supplementary file 6 — Additional file 6. Metanalyses of indirect outcomes. [file 13018_2022_3040_MOESM6_ESM.docx]

Article title: Transtibial versus Independent Femoral Tunnel Drilling Techniques for Anterior Cruciate Ligament reconstruction: Evaluation of Femoral Aperture Positioning. A Systematic review and Meta-analysis

Journal name: Journal of Orthopaedic Surgery and Research

Author names and affiliation: Haitham K. Haroun^1^, Maged M. Abouelsoud^1^, Mohamed R. Allam ^2^, and Mahmoud M. Abdelwahab^1^

^1^ Orthopedic Department, Faculty of Medicine, Ain Shams University, Cairo, Egypt

^2^El Demerdash Hospital, Ain-Shams University, Cairo, Egypt

e-mail address of the corresponding author: haroun.haitham@med.asu.edu.eg

**Additional file 6: Metanalyses of indirect outcomes**


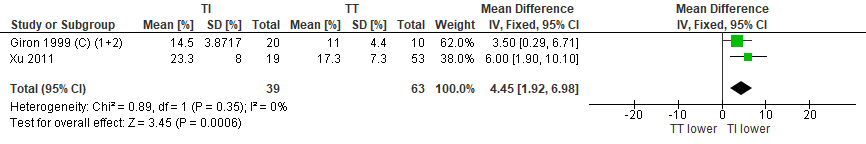


Fig. a: Aperture position perpendicular to BL by quadrant method on radiograph


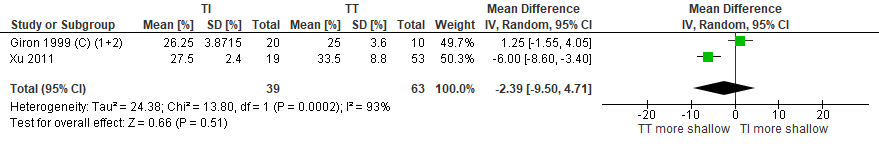


Fig. b: aperture position along BL by quadrant method on radiograph


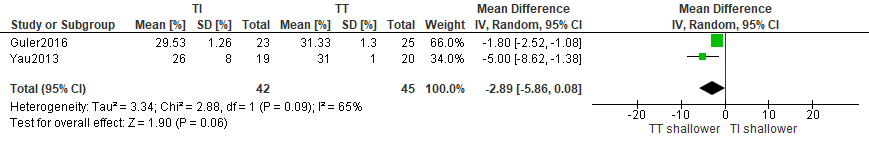


Fig. c: Aperture position along BL by quadrant method on MRI


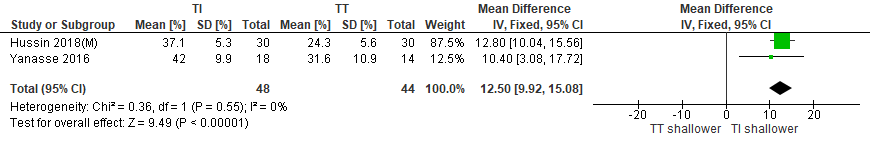


Fig. d: Aperture position% from whole BL by Harner method radiograph
