# Supplementary material for: Transtibial versus independent femoral tunnel drilling techniques for anterior cruciate ligament reconstruction: evaluation of femoral aperture positioning
Source: J Orthop Surg Res. 2022 Mar 18;17:166. doi: 10.1186/s13018-022-03040-5 (PMC8931956; doi:10.1186/s13018-022-03040-5)
Supplement: Supplementary file 7 — Additional file 7. Metanalyses of tibial aperture position. [file 13018_2022_3040_MOESM7_ESM.docx]

Article title: Transtibial versus Independent Femoral Tunnel Drilling Techniques for Anterior Cruciate Ligament reconstruction: Evaluation of Femoral Aperture Positioning. A Systematic review and Meta-analysis

Journal name: Journal of Orthopaedic Surgery and Research

Author names and affiliation: Haitham K. Haroun1, Maged M. Abouelsoud1, Mohamed R. Allam 2, and Mahmoud M. Abdelwahab1

^1^ Orthopedic Department, Faculty of Medicine, Ain Shams University, Cairo, Egypt

^2^El Demerdash Hospital, Ain-Shams University, Cairo, Egypt

e-mail address of the corresponding author: haroun.haitham@med.asu.edu.eg

Additional file 5: Metanalyses of tibial aperture position


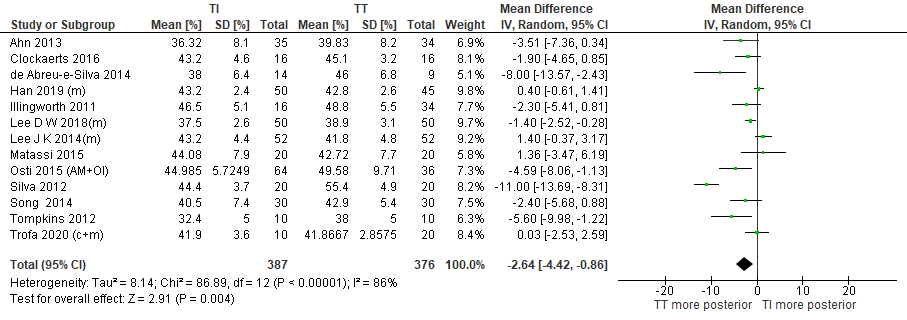


Tibial aperture AP position measured on CT (Higher percentage is defined as more posterior aperture location)


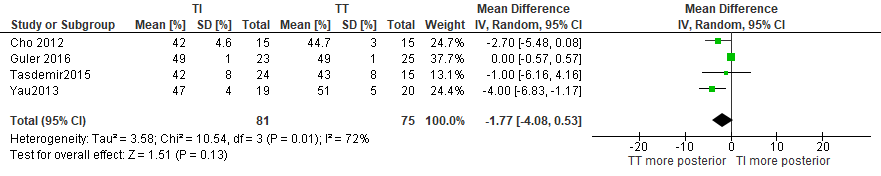


Tibial aperture AP position measured on MRI (Higher percentage is defined as more posterior aperture location)


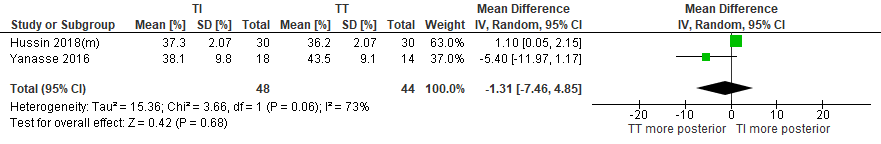


Tibial aperture AP position measured on radiography (Higher percentage is defined as more posterior aperture location)


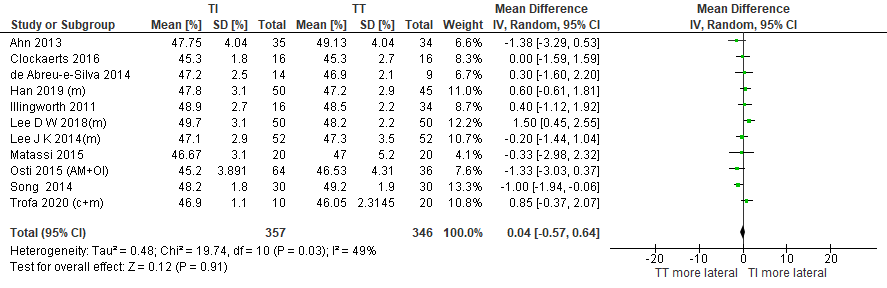


Tibial aperture ML position measured on CT (Higher percentage is defined as more lateral aperture location).
