# Supplementary material for: Transtibial versus independent femoral tunnel drilling techniques for anterior cruciate ligament reconstruction: evaluation of femoral aperture positioning
Source: J Orthop Surg Res. 2022 Mar 18;17:166. doi: 10.1186/s13018-022-03040-5 (PMC8931956; doi:10.1186/s13018-022-03040-5)
Supplement: Supplementary file 8 — Additional file 8. Subgroup analyses by variation in TI techniques {AM vs OI} (Fig. A) and by modifications to TT technique {conventional TT vs modified TT (mTT)} (Fig. B). [file 13018_2022_3040_MOESM8_ESM.docx]

Article title: Transtibial versus Independent Femoral Tunnel Drilling Techniques for Anterior Cruciate Ligament reconstruction: Evaluation of Femoral Aperture Positioning. A Systematic review and Meta-analysis

Journal name: Journal of Orthopaedic Surgery and Research

Author names and affiliation: Haitham K. Haroun^1^, Maged M. Abouelsoud^1^, Mohamed R. Allam ^2^, and Mahmoud M. Abdelwahab^1^

^1^ Orthopedic Department, Faculty of Medicine, Ain Shams University, Cairo, Egypt

^2^El Demerdash Hospital, Ain-Shams University, Cairo, Egypt

e-mail address of the corresponding author: haroun.haitham@med.asu.edu.eg

**Additional file 8:** **(Fig. a) Subgroup analysis by variation in TI techniques {AM vs OI} in Femoral aperture position perpendicular to BL measured by quadrant method on CT**

**
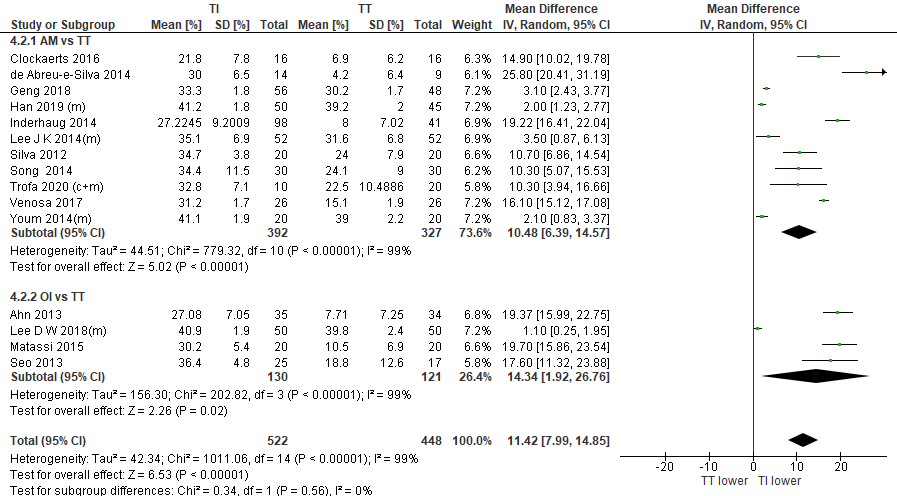
**

(m) modified TT, (c+m) combined conventional and modified TT groups.

Subgroup difference is non-significant (P=0.56)

**Additional file 8: (Fig. b) Subgroup analysis by modifications to TT technique {conventional TT (cTT) vs modified TT (mTT)} in Femoral aperture position perpendicular to BL measured by quadrant method on CT**


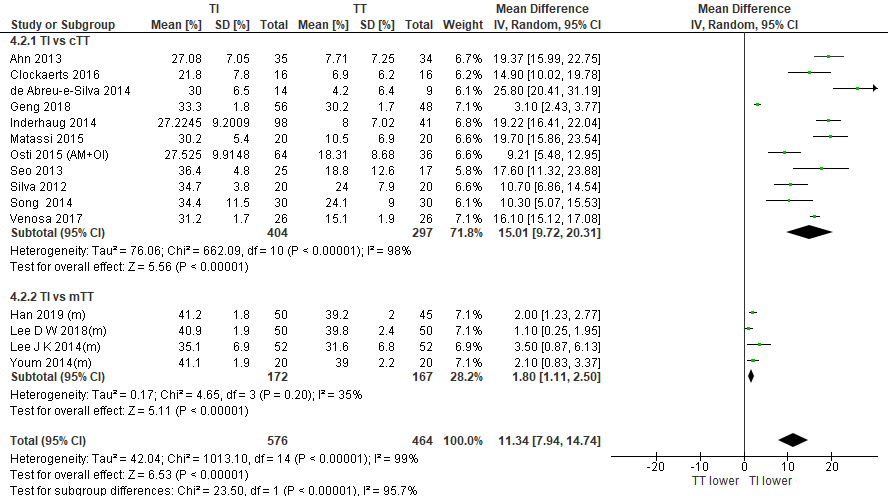


(AM+OI) combined AM and OI groups

Subgroup difference is significant (P< 0.00001). Modifications to TT technique had an effect of 13% on the aperture position perpendicular to BL (decreased the mean difference from 15 % to 1.8 %).
